# Transcriptomic subtyping of malignant peripheral nerve sheath tumours highlights immune signatures, genomic profiles, patient survival and therapeutic targets

**DOI:** 10.1016/j.ebiom.2023.104829

**Published:** 2023-10-12

**Authors:** Maren Høland, Kaja C.G. Berg, Ina A. Eilertsen, Bodil Bjerkehagen, Matthias Kolberg, Kjetil Boye, Ole Christian Lingjærde, Tormod K. Guren, Nils Mandahl, Eva van den Berg, Emanuela Palmerini, Sigbjørn Smeland, Piero Picci, Fredrik Mertens, Anita Sveen, Ragnhild A. Lothe

**Affiliations:** a Department of Molecular Oncology, Institute for Cancer Research, Oslo University Hospital, Oslo, Norway; b Institute for Clinical Medicine, University of Oslo, Oslo, Norway; c Division of Laboratory Medicine, Department of Pathology, Oslo University Hospital, Oslo, Norway; d Division of Cancer Medicine, Department of Oncology, Oslo University Hospital, Oslo, Norway; e Department of Informatics, Faculty of Mathematics and Natural Sciences, University of Oslo, Oslo, Norway; f Department of Clinical Genetics, University and Regional Laboratories, Lund University, Lund, Sweden; g Department of Genetics, The University Medical Center Groningen, the Netherlands; h Osteoncology, Bone and Soft Tissue Sarcomas and Innovative Therapies, IRCCS Istituto Ortopedico Rizzoli, Bologna, Italy; i Laboratory of Experimental Oncology, IRCCS Istituto Ortopedico Rizzoli, Bologna, Italy

**Keywords:** MPNST, Transcriptomic subtypes, DNA copy number aberrations, Data integration, Prognosis

## Abstract

**Background:**

Malignant peripheral nerve sheath tumour (MPNST) is an aggressive orphan disease commonly affecting adolescents or young adults. Current knowledge of molecular tumour biology has been insufficient for development of rational treatment strategies. We aimed to discover molecular subtypes of potential clinical relevance.

**Methods:**

Fresh frozen samples of MPNSTs (n = 94) and benign neurofibromas (n = 28) from 115 patients in a European multicentre study were analysed by DNA copy number and/or transcriptomic profiling. Unsupervised transcriptomic subtyping was performed and the subtypes characterized for genomic aberrations, clinicopathological associations and patient survival.

**Findings:**

MPNSTs were classified into two transcriptomic subtypes defined primarily by immune signatures and proliferative processes. “Immune active” MPNSTs (44%) had sustained immune signals relative to neurofibromas, were more frequently low-grade (*P* = 0.01) and had favourable prognostic associations in a multivariable model of disease-specific survival with clinicopathological factors (hazard ratio 0.25, *P* = 0.003). “Immune deficient” MPNSTs were more aggressive and characterized by proliferative signatures, high genomic complexity, aberrant *TP53* and PRC2 loss, as well as high relative expression of several potential actionable targets (*EGFR, ERBB2, EZH2, KIF11, PLK1, RRM2*). Integrated gene-wise analyses suggested a DNA copy number-basis for proliferative transcriptomic signatures in particular, and the tumour copy number burden further stratified the transcriptomic subtypes according to patient prognosis (*P* < 0.01).

**Interpretation:**

Approximately half of MPNSTs belong to an “immune deficient” transcriptomic subtype associated with an aggressive disease course, PRC2 loss and expression of several potential therapeutic targets, providing a rationale for molecularly-guided intervention trials.

**Funding:**

Research grants from non-profit organizations, as stated in the Acknowledgements.

**Research in context**

**Evidence before this study**

MPNST is a highly aggressive cancer type and there are few effective treatment options for patients with unresectable tumours. Most patients are adolescents or young adults. The MPNST genome is complex. Specific cancer-critical target genes have frequent copy number gains or losses, and mutational inactivation of PRC2 results in aberrant transcriptional activation of several developmental pathways. However, the impact of genomic aberrations on the tumour transcriptome is not well described in general. Molecular knowledge currently has no impact on the treatment of patients. The rarity of the disease is a challenge for translational research, and most molecular studies have included few patients and tumours.

**Added value of this study**

This study presents the molecular profiles of a collection of MPNSTs from four European sarcoma centres. We showed that transcriptomic immune or proliferative signals and DNA copy number complexity represent correlated and discriminatory features of MPNSTs. The tumours can be divided into two transcriptomic subtypes associated with large differences in the survival rates of the patients, independently of clinicopathological factors. The most aggressive subtype was deficient of immune signals and had high relative genomic complexity, including loss of PRC2 and copy number-driven expression of proliferative signatures. The least aggressive subtype had retained immune signals relative to benign neurofibromas and was enriched with low-grade malignant tumours. The subtypes were independent of the hereditary syndrome neurofibromatosis type 1, although hereditary malignant tumours appeared most distinct from benign neurofibromas across data levels.

Although distinct subtypes were found, the discriminatory molecular features were of a continuous rather than discrete nature across the tumours and subtype boundaries. Consistently, the DNA copy number burden was a prognostic factor also within each of the transcriptomic subtypes. Several potential drug targets showed consistent regulation at the DNA copy number and gene expression levels, and were distinct for each of the transcriptomic subtypes.

**Implications of all the available evidence**

This study adds to the growing body of evidence for the role of PRC2 loss in shaping the molecular biology of a subgroup of approximately half of all MPNSTs associated with an aggressive disease course. The integrated molecular map of cancer-critical genes and therapeutic targets on the DNA copy number and transcriptomic levels presents further opportunity for drug development studies in MPNSTs.

## Introduction

Malignant peripheral nerve sheath tumours (MPNSTs) are rare and highly aggressive cancers that predominantly occur among adolescents and young adults.^1^ The tumours arise from neural crest-derived cells, either sporadically or in association with the hereditary syndrome neurofibromatosis type 1 (NF1), which is caused by a germline mutation in the tumour suppressor gene *NF1*. The incidence of MPNST is high among patients with NF1,^2^ and approximately half of the cancers occur in this hereditary setting.^1^ The outcome of patients with MPNST is poor independent of NF1-status,^1^ and less than 50% survive five years after diagnosis. The tumours are often insensitive to existing chemotherapies and radiotherapy, and complete surgical resection is the only potentially curative treatment. However, resected MPNSTs often relapse and complete tumour resection is not possible in all patients due to a large tumour size, the tumour location and/or metastasis at the time of diagnosis.^1^^,^^3^ Chemotherapies targeting DNA topoisomerase II are frequently used in both the adjuvant and metastatic settings.^4^^,^^5^ A phase II study of doxorubicin and etoposide in combination with ifosfamide for high-grade or metastatic MPNSTs showed a partial response in 24% and stable disease in 70% of the 37 evaluable patients.^6^ However, the efficacy of etoposide in relation to the expression of DNA topoisomerase II components has not been evaluated. No molecularly-guided treatment strategies are currently available, and improved molecular knowledge is needed to develop rational treatment strategies against this orphan malignancy.

The genomes of MPNSTs are characterized by a large number of recurrent DNA copy number aberrations (CNAs).^7^, ^8^, ^9^ The tumour suppressor gene *CDKN2A* is the target of frequent deletions on chromosome arm 9p.^10^, ^11^, ^12^, ^13^ This event occurs early in MPNST development, and has been found also in benign and atypical neurofibromas. *NF1* is frequently targeted by deletion of the proximal part of chromosome arm 17q, and is often co-deleted with *SUZ12*, which encodes a core component of the polycomb repressive complex 2 (PRC2).^14^ *NF1* and *SUZ12* are also among the few identified targets of recurrent mutations at the nucleotide level, together with *TP53* and *EED* (encoding another PRC2 component).^14^, ^15^, ^16^, ^17^, ^18^ Loss of function of PRC2 results in epigenetic deregulation by loss of trimethylation at lysine 27 of histone H3 (H3K27me3), and might be associated with malignant progression of the tumours.^14^^,^^18^ DNA copy number gains frequently occur at chromosome arms 7p and the distal part of 17q, and include *EGFR*, *TOP2A*, and *BIRC5* as the proposed targets.^19^, ^20^, ^21^, ^22^, ^23^

The impact of genomic aberrations on the transcriptomes of MPNSTs is not well described, with the notable exception of aberrant transcriptional activation of PRC2-repressed homeobox master regulators in tumours with PRC2 loss.^14^ There are few consistent differences in gene expression between NF1-associated and sporadic MPNSTs.^24^^,^^25^ Gene expression studies have been few and limited by a small sample size (10–45 tumours), narrow coverage, or lack of prognostic data.^7^^,^^24^, ^25^, ^26^, ^27^, ^28^ However, a recent study of 90 tumours by the Genomics of MPNST Consortium supported the stratification of MPNST transcriptomes (and methylomes) according to H3K27me3 status.^16^ We have previously proposed that a gene expression-based phenotype of aberrant *TP53* is associated with a poor outcome of the patients.^17^ In this study, we aimed to discover molecular subtypes of potential clinical relevance. We hypothesized that the genomic complexity of MPNSTs is reflected in the transcriptome, and analysed MPNSTs and neurofibromas from 115 patients treated at four European sarcoma centres by genome-wide DNA copy number and/or gene expression profiling.

## Methods

### Patients and samples

The patients were treated at four European sarcoma centres between 1980 and 2010, including the Norwegian Radium Hospital, Oslo, Norway; Skåne University Hospital, Lund, Sweden; the University Medical Centre of Groningen, The Netherlands; and the Istituto Ortopedico Rizzoli, Bologna, Italy. Fresh frozen tissue samples were available from surgical specimens of 94 MPNSTs (Supplementary Table S1) and 28 cutaneous neurofibromas (only one recorded as a plexiform tumour, Supplementary Table S2) from a total of 115 patients, including matched sample pairs from seven patients. Patients with both NF1-associated and sporadic MPNSTs were included, and the representation of sexes was fairly balanced (Table 1). Sex was self-reported by the study participants and not considered in the study design. None of the MPNSTs were radiation-induced.

**Table 1:** Clinical parameters of patients and MPNSTs.

Variable	DNA copy number analysis	Gene expression analysis	Transcriptomic subtypes		
			Immune active	Immune deficient	Odds ratio [95% CI] (Fisher's exact test)
	Number of patients		Number of patients		
All	93	64	28	36	
Overlapping between methods	63				
Neurofibromatosis type 1					
No	46 (49%)	32 (50%)	15 (54%)	17 (47%)	1.3 [0.4–3.9], *P* = 0.8
Yes	47 (51%)	32 (50%)	13 (46%)	19 (53%)	
Sex					
Female	41 (44%)	29 (45%)	15 (54%)	14 (39%)	1.8 [0.6–5.6], *P* = 0.3
Male	52 (56%)	35 (55%)	13 (46%)	22 (61%)	
Age at diagnosis					
Below median (≤36 years)	47 (51%)	32 (50%)	11 (39%)	21 (58%)	0.5 [0.2–1.4], *P* = 0.2 (linear variable, Wilcoxon: *P* = 0.25)
Above median (>36 years)	46 (49%)	32 (50%)	17 (61%)	15 (42%)	
Tumour location					
Extremities	67 (72%)	46 (72%)	21 (75%)	25 (69%)	1.3 [0.4–4.8], *P* = 0.8
Non-extremities	26 (28%)	18 (28%)	7 (25%)	11 (31%)	
Tumour size^a^					
Below median (≤11 cm)	45 (48%)	31 (48%)	17 (61%)	14 (39%)	2.6 [0.9–8.5], *P* = 0.08 (linear variable, Wilcoxon: *P* = 0.03)
Above median (>11 cm)	42 (45%)	32 (50%)	10 (36%)	22 (61%)	
Not available	6 (6%)	1 (2%)	1 (4%)	0 (0%)	
Complete remission^b^					
No	16 (17%)	12 (19%)	4 (14%)	8 (22%)	0.6 [0.1–2.7], *P* = 0.5
Yes	61 (66%)	51 (80%)	23 (82%)	28 (78%)	
Not available	16 (17%)	1 (2%)	1 (4%)	0 (0%)	
Grade of primary tumour^c^					
Low	7 (8%)	5 (8%)	5 (18%)	0 (0%)	Inf [1.3-Inf], *P* = 0.01
High	84 (90%)	59 (92%)	23 (82%)	36 (100%)	
Not available	2 (2%)	0 (0%)	0 (0%)	0 (0%)	
Metastasis at time of diagnosis					
No	82 (88%)	56 (88%)	24 (86%)	32 (89%)	0.8 [0.1–6.1], *P* = 1
Yes	8 (9%)	6 (9%)	3 (11%)	3 (8%)	
Not available	3 (3%)	2 (3%)	1 (4%)	1 (3%)	
Tumour analysed					
Primary	69 (74%)	48 (75%)	21 (75%)	27 (75%)	1 [0.3–3.7], *P* = 1
Relapse	24 (26%)	16 (25%)	7 (25%)	9 (25%)	

CI, confidence interval; Inf, infinity.

a Maximum diameter of the primary tumour.

b Wide or marginal surgical margins after removal of the primary tumour and no metastasis at diagnosis.

c Grade of the analysed tumour (relapse or second primary) is given for five patients (three high-grade, two low-grade).

The MPNST diagnosis was determined by sarcoma pathologists at the sarcoma referral centres in each country, according to established criteria.^29^^,^^30^ The diagnosis was re-examined and confirmed in neighbouring tissue sections of all fresh frozen tumour samples, and the tumour content was visually estimated to a median of ∼100% (interquartile range: 14%). DNA and RNA have previously been extracted, and only RNA samples with an RNA integrity number above 6 were analysed (evaluated on an Agilent 2100 Bioanalyzer, Agilent Technologies, Santa Clara, CA, U.S.A.). Evaluation of gene expression for *S100B, S100A1,* and *SOX10* supported the correct diagnosis of MPNSTs.^17^ DNA from white blood cell samples from 18 of the patients was extracted by a magnetic beads protocol (Maxwell 16 DNA purification kit, Promega, Madison, WI, U.S.A.).

Tumours were analysed for specific CNAs and the total burden of CNAs. Subtype discovery of MPNSTs was performed on the transcriptomic level, and subtypes were analysed for genomic aberrations, gene set enrichments and clinicopathological associations, as summarized in the flow chart in Supplementary Fig. S1. Integration analysis of CNAs and the expression level of each targeted gene was focused on genes with differential expression between the transcriptomic subtypes, cancer-critical genes and genes encoding drug targets.

### Ethics

The biobanks and research protocols were approved by the regional/local ethics committees for each participating hospital, following informed consent from the patients. The multicentre analyses performed in this study were approved by the Regional Committee for Medical and Health Research Ethics South-Eastern Norway (2010/223/REKsør-øst).

### High resolution DNA copy number analyses

Genomic DNA from 93 of the 94 MPNSTs and 28 neurofibromas was analysed on genome-wide Human SNP Arrays 6.0 according to the manufacturer's instructions (Thermo Fisher Scientific, Waltham, MA, U.S.A.), and as previously described. Microarrays were preferred over shallow whole-genome sequencing for DNA copy number estimation, to obtain sufficient analysis depth in the potentially heterogeneous tumours. Filtration of germline variants was performed against a large reference pool of normal samples from the HapMap project and a custom collection of normal samples, as previously described.^17^^,^^31^ Gene-level copy number estimates were retrieved as described in Supplementary Methods and Supplementary Table S3, and 18,091 autosomal protein-coding genes were successfully mapped to the segmented data.

DNA copy number gains and losses were called from data segmented by the PCF algorithm.^32^ Allele-specific data were called with the ASCAT algorithm^33^ and used to estimate tumour ploidy and the cancer cell fraction, as well as to identify genes with amplification or high-level amplification (gain of ≥5 or ≥10 copies, respectively), homozygous loss, and loss of heterozygosity (LOH), but were not scored for two of the MPNSTs. A methodological comparison of the PCF and ASCAT algorithms showed a strong correlation in the estimated DNA copy number gains and losses separately across chromosomes (Spearman's ρ = 0.96 and ρ = 0.97) and across the genome of individual MPNSTs (ρ = 0.88 and ρ = 0.88, Supplementary Fig. S2). Tumour ploidy ranged from 1n to 5n among MPNSTs (Supplementary Fig. S3), while all neurofibromas and white blood cell samples were diploid. The triploid MPNSTs had a higher percentage of CNAs (median = 48%) than both the diploid (median = 13%, *P* = 1 × 10^−12^) and the tetraploid MPNSTs (median = 25%, *P* = 0.05, both from Wilcoxon's test).

### Gene expression profiling

Total RNA (100 ng) from 64 MPNSTs (Table 1 and Supplementary Table S1) and 15 neurofibromas (including the plexiform tumour; Supplementary Table S2) was analysed by gene expression profiling on GeneChip Human Transcriptome 2.0 Arrays (Thermo Fisher Scientific) as previously described.^17^ In short, arrays were run according to the manufacturer's instructions and CEL files were background corrected, quantile normalized and summarized at the gene-level using the Signal Space Transformation and Robust Multi-array Average (SST-RMA) approach implemented in the Affymetrix Expression Console Software (v1.4.1.46, HTA-2.0_0.r3 library files), estimating gene expression data on a log2-scale for 18,567 protein-coding genes. We have previously shown a strong correspondence between these microarrays and RNA sequencing for gene expression estimation in solid tumours.^34^

Differential gene expression analyses, unsupervised subtype discovery and principal component analysis (PCA) were performed as described in Supplementary Methods. Gene set enrichment analyses of the Hallmark gene sets (n = 50) and gene sets for the PRC2 complex^14^ and TP53 signalling^35^ between sample groups were performed by the camera gene set test implemented in the limma package.^36^ The Hallmark gene sets represent a collection of curated gene sets of biological states and processes, and were downloaded from the Molecular Signatures Database (https://www.gsea-msigdb.org/gsea/msigdb/human/genesets.jsp? collection = H; accessed April 10, 2018).^37^ Gene set variation analysis was used to estimate single-sample enrichment (ssGSVA) scores using the R package GSVA (v1.22.4) with default settings.^38^

### Integration of DNA copy number and gene expression data

Both DNA copy number and gene expression data were available for 63 MPNSTs and 17,618 autosomal protein-coding genes, of which 7490 (43%) had expression variance >0.3 across the dataset and were included in the integration analyses. Concurrent gain and upregulated gene expression or loss and downregulated gene expression was called for genes with differential expression between samples with neutral copy number and gain or loss, respectively (false discovery rate adjusted P-value [FDR*-P*]< 0.05 from Wilcoxon rank-sum test and median gene expression difference >|0.5|). The minimum number of samples with gain or loss was set to 5, and a positive correlation between DNA copy number and gene expression was required (ρ > 0 and FDR-*P*< 0.05 from Spearman's correlation analysis using the raw PCF estimates as input).

### Gene annotations

All genomic positions refer to genome version GRCh37 (Hg19). Genes were considered cancer-critical if included in the COSMIC Cancer Gene Census (v83: https://cancer.sanger.ac.uk/census) or among known MPNST relevant genes (Supplementary Table S4) or genes encoding drug targets (Supplementary Table S5). The list of drug targets was compiled from cancer treatments in the online DrugBank database (http://www.drugbank.ca; accessed October 2017), excluding targets of conventional chemotherapeutics.^39^

### Statistics and survival analyses

General statistical analyses were performed using functions in R (https://www.R-project.org/). Fisher's exact test (fisher.test, two-sided unless stated otherwise) was used to compare frequency distributions between groups by the odds ratio (OR), Wilcoxon rank-sum test (wilcox.test) was used for continuous variables in two-group comparisons, and Spearman's test (cor.test) was used to analyze correlation for continuous and ordinal variables. *P*-values were adjusted for multiple testing with FDR using the p.adjust function.

Patients with samples from a primary MPNST or a local relapse were included for survival analyses, except three patients who were lost to follow-up (total n = 86; Supplementary Table S1 and Supplementary Fig. S1). Five-year disease-specific survival was estimated from the time of diagnosis of the primary MPNST, and death from MPNST was considered an event. Patients with no events within five years were censored (n = 9). Univariable and multivariable survival analyses were performed by Cox proportional regression using the R package survival (function coxph) to calculate hazard ratios (HRs) and 95% confidence intervals (CIs), with *P*-values derived from Wald test. Multivariable analyses were performed with the clinicopathological variables age at diagnosis, complete remission, NF1-status, sex, tumour site and size, and patients without all information available were excluded (n = 12, 92% due to missing remission status). Kaplan–Meier survival curves were estimated and plotted with the R package survminer (functions survfit and ggsurvplot).

### Role of funders

The study was funded by internal hospital budgets and molecular analyses by external grants from the Norwegian Cancer Society and the South-Eastern Norway Regional Health Authority. The funders had no role in the study design, data collection, data analysis, interpretation, or writing of the manuscript.

## Results

### Copy number aberrations are frequent and diverse among MPNSTs

DNA copy number profiles were initially compared across a multicentre collection of MPNSTs with (n = 47) or without (n = 46) a hereditary component (Table 1 and Supplementary Table S1) and benign neurofibromas (n = 28; Supplementary Table S2). The neurofibromas were cutaneous and only one was recorded as plexiform. These tumours do not undergo malignant transformation and were analysed as a reference for non-malignant peripheral nerve tissue. The benign tumours had a few recurrent events, including loss of *NF1* and *SUZ12* on chromosome arm 17q, as well as *CDKN2A, CDKN2B* and *MTAP* on 9p (*q* < 1 × 10^−4^ from GISTIC analysis^40^; Supplementary Table S6). The *CDKN2A* locus (9p21.3) was identified as the only target of recurrent homozygous deletions in both neurofibromas (n = 4, 14%, including the plexiform tumour) and MPNSTs (44%; Supplementary Fig. S4). Previous studies have shown conflicting results with respect to the presence of genomic aberrations on 9p in neurofibromas, potentially related to histopathological differences of the tumours or small sample sizes in some of the studies.^10^^,^^14^^,^^41^^,^^42^

**Fig. 1:**
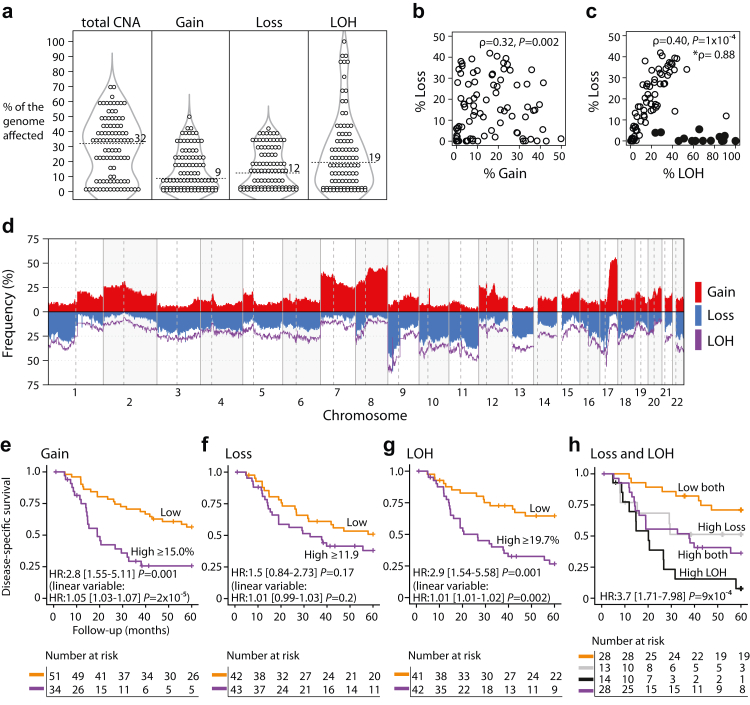
**Genome-wide copy number aberrations across MPNSTs. (a)** Percentage of the genome affected by DNA copy number gain, loss and both (total CNA; n = 93), as well as LOH (n = 91). Number at dashed line is the median value. Scatter plots show the association between **(b)** loss and gain, and **(c)** loss and LOH. Filled circles (black) indicate tumours (n = 14) with a disproportionate percentage of LOH and loss. Statistics are from Spearman's rank correlation and the asterisk indicates the correlation coefficient after exclusion of tumours with filled circles. **(d)** Frequency of genome-wide gains, losses, and LOH among tumours. **(e**–**g)** Kaplan–Meier survival curves for patients with MPNSTs with high and low levels of copy number gain, loss and LOH (see Supplementary Fig. S8a for dichotomization of the tumours). Hazard ratios and 95% CIs (in brackets) are from Cox regression analyses and *P*-values from Wald tests. Results from analysis of the linear variables (percentage aberration) are given in parentheses. **(h)** Kaplan–Meier survival curves for the combined variable of loss and LOH, grouped according to panels (f) and (g). Hazard ratio was calculated by comparing patients with low percentage of genome-wide loss and LOH (orange) versus the combined group of patients with high percentage of loss and/or LOH (grey, purple, black).

MPNSTs had a much higher genomic complexity than neurofibromas, estimated as the proportion of the genome affected by CNAs (median 32% versus 0.04%, *P* = 2 × 10^−11^ from Wilcoxon's test; Fig. 1a and Supplementary Fig. S5). There was a large variation in the CNA burden across MPNSTs, and the percentage of the genome affected ranged from 0 to 70% (10th-90th percentile 0.5%–57%; Fig. 1a). The CNA profiles were similar between the NF1-associated and sporadic tumours, although there was a trend towards a higher burden of CNAs (*P* = 0.07), LOH (*P* = 0.05, both from Wilcoxon's test), and homozygous deletion of *CDKN2A* (OR = 2.4, *P* = 0.06 from Fisher's exact test) in NF1-associated tumours (Supplementary Fig. S6).

There was little correspondence in the burden of DNA copy number gains and losses among MPNSTs (Fig. 1b). Gains most recurrently affected chromosome arms 7p, 8q, and the distal part of 17q (56%, 47%, and 41% of MPNSTs, respectively), all of which were also commonly affected by focal amplifications (≥5 additional copies; Supplementary Table S3 and Supplementary Fig. S7). Half (n = 46, 51%) of MPNSTs had amplification of at least one protein-coding gene, and 19% (n = 17) had a high-level amplification (≥10 additional copies), commonly also of a cancer-critical gene (n = 14/17, 82%; Supplementary Tables S1 and S7). DNA copy number losses were typically accompanied by a proportional amount of LOH, both in individual MPNSTs (Fig. 1c) and across the genome (Fig. 1d). These aberrations were most frequently found on chromosome arms 9p, 11q, 17p (61%, 37%, and 41% of MPNSTs, respectively), and the proximal part of 17q (38%). Notably, a small subgroup of MPNSTs (n = 14, 15%) showed disproportionate profiles, with a large part of the genome affected by LOH (range 24%–100%) and a low burden of copy number losses (<6%; Fig. 1c).

### The genomic complexity of MPNSTs has prognostic impact

A large CNA burden was associated with an inferior disease-specific survival among patients with MPNST (Fig. 1e and f and Supplementary Fig. S8). The prognostic association was stronger for DNA copy number gains than losses, and the burden of gains had independent prognostic value in a multivariable analysis including NF1-status, age, sex, and remission status of the patients, as well as tumour location and size (burden of gain analysed as a continuous variable: HR = 1.04, 95% CI 1.01–1.08, *P* = 0.004 from Wald test; Supplementary Table S8). Several genes with frequent DNA copy number gains and amplifications were markers of a poor prognosis (Supplementary Table S3, Supplementary Fig. S7c–h and Supplementary Fig. S8d).

The tumour burden of LOH was also associated with a poor survival among the patients (Fig. 1g), including in the multivariable model (HR = 1.02, 95% CI 1.003–1.03, *P* = 0.02 from Wald test; Supplementary Table S8). Combined analysis of DNA copy number losses and LOH further improved the prognostic stratification (Fig. 1h). Patients with a low burden of both aberration types had a five-year disease-specific survival rate of 71%, compared to 30% for patients with high amounts of losses and/or LOH combined. The subgroup with a disproportionate amount of LOH and copy number losses (LOH high, loss low; Fig. 1c) had the lowest survival rate (7.7%; Fig. 1h). The prognostic value of the different CNA estimates (burden of copy number gain, loss combined with LOH) were independent in a bivariable analysis (high versus low gain: HR = 2.4, 95% CI 1.3–4.4, *P* = 0.005; high loss and/or high LOH versus low both: HR = 3.2, 95% CI 1.5–7.1, *P* = 0.003 from Wald test).

### Transcriptomic subtypes of MPNSTs based on immune signatures and proliferative processes

A subset of the MPNSTs (n = 64) and neurofibromas (n = 15; including the plexiform tumour) were also analysed by gene expression profiling. The MPNSTs and non-malignant reference samples were clearly separated along both of the two first axes in PCA (*P* = 2 × 10^−8^ and *P* = 3 × 10^−3^ from Wilcoxon's test; Supplementary Fig. S9a), and multiple cancer-critical genes were differentially expressed, including *TOP2A* (Supplementary Fig. S9b). Gene set enrichment analysis of the Hallmark gene set collection (n = 50)^37^ showed downregulation of immunological processes in the malignant tumours, including interferon-α and interferon-γ response (FDR-*P*<1 × 10^−8^), and upregulation of proliferative processes, including the E2F and MYC targets and the G2M checkpoint (FDR-*P*<1 × 10^−9^, both from camera gene set test^36^; Supplementary Table S9). Sporadic MPNSTs clustered between the benign neurofibromas and the NF1-associated MPNSTs in the two-dimensional PCA (Supplementary Fig. S9a). The two MPNST groups were not separated along the first principal component (*P* = 0.5), but showed a clear distinction along the second (*P* = 0.01, both from Wilcoxon's test). However, *PCDH9* was the only differentially expressed gene (fold-change 1.6, FDR-*P* = 2 × 10^−4^ from limma analysis^43^; Supplementary Fig. S9), and both MPNST groups showed enrichment with the same gene sets relative to neurofibromas (Supplementary Table S9).

**Fig. 2:**
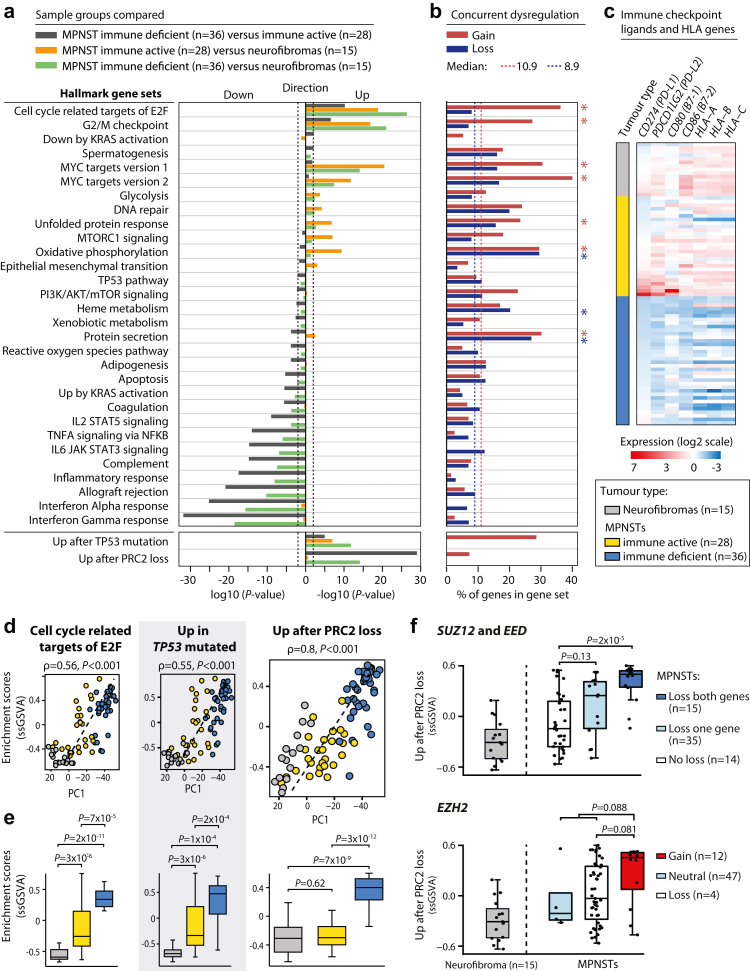
**Gene expression characteristics of the transcriptomic MPNST subtypes. (a)** Gene set enrichment analyses of the Hallmark (n = 50), TP53 and PRC2 gene sets between MPNST transcriptomic subtypes and benign neurofibromas as indicated. Gene sets with FDR-corrected *P* < 0.01 from camera gene set test were included for plotting, and the Hallmark gene sets were ordered according to significance levels from comparison of the two MPNST subtypes. **(b)** Proportion of genes per gene set with concurrent gain and upregulated expression (red), or loss and downregulated expression (blue) among MPNSTs (n = 63). Vertical dashed lines indicate the median number of concurrent genes among the 50 Hallmark gene sets, and asterisks indicate gene sets with a significantly larger number of concurrent genes than the median (evaluated by one-sided Fisher's tests). **(c)** Expression levels of selected immune genes in MPNSTs and neurofibromas (mean-centred and scaled to standard deviation 1). Tumours are sorted according to transcriptomic subtype and the expression level of *CD274*. Single-sample enrichment (ssGSVA) scores of selected gene sets plotted **(d)** against PC1 from principal component analysis of tumours (dashed line shows the linear regression); **(e)** according to tumour type and coloured as in panel (c); and **(f)** according to DNA copy number status of PRC2 core components. Statistics are from Spearman's rank correlation in panel (d) (calculated among the 64 MPNSTs only, neurofibromas were included for illustration), and Wilcoxon's test in panels (e) and (f).

Unsupervised gene expression-based classification of MPNSTs by non-negative matrix factorization indicated that the malignant tumours can be divided into two transcriptomic subtypes (Supplementary Fig. S10a and b). The subtypes were independent of the NF1-status of the patients (Table 1). Gene set enrichment analyses showed strong downregulation of immune-related processes in the largest MPNST subtype (56% of tumours) relative to the smallest (44%) and relative to neurofibromas (Fig. 2a). The largest subtype also had low relative expression levels of all genes encoding human leukocyte antigens (n = 19 genes with prefix “HLA”; Supplementary Table S10), as well as the PD-1 and CTLA-4 checkpoint ligands, and was termed immune deficient (Fig. 2c). The smallest MPNST subtype showed a similar level of immune activity relative to the non-malignant samples and was termed immune active. Notably, the level of immune activity appeared to be a continuous rather than a discrete feature among MPNSTs, also within the two subtypes (Supplementary Fig. S10c). A particularly high expression level of genes encoding immune checkpoint ligands was observed in a subset of the immune active MPNSTs (Fig. 2c).

The immune deficient subtype showed enrichment with gene sets related to cell cycle progression compared to both immune active MPNSTs and neurofibromas separately, including targets of E2F and the G2/M checkpoint (Fig. 2a). These processes also had a continuous change in expression patterns within and across the tumour groups (Fig. 2d and e). Gene sets of particular relevance to MPNSTs were strongly enriched in the immune deficient subtype, including mutated *TP53* and loss of activity of PRC2.^14^^,^^17^ The signature for loss of PRC2 activity was associated with the CNA status of PRC2 core components, and the highest signature scores were found in MPNSTs with loss of *SUZ12* and *EED*, or gain of *EZH2* (Fig. 2f). Loss of PRC2 activity appeared to be specific to the immune deficient subtype (Fig. 2a and e), and these MPNSTs were enriched with CNAs of all three core component genes compared to immune active MPNSTs (OR = 3.8, *P* = 0.03; OR = 5.5, *P* = 0.007; OR = 5.1, *P* = 0.051, respectively, all from Fisher's exact test).

### Transcriptomic subtypes are linked to DNA copy number aberrations

**Fig. 3:**
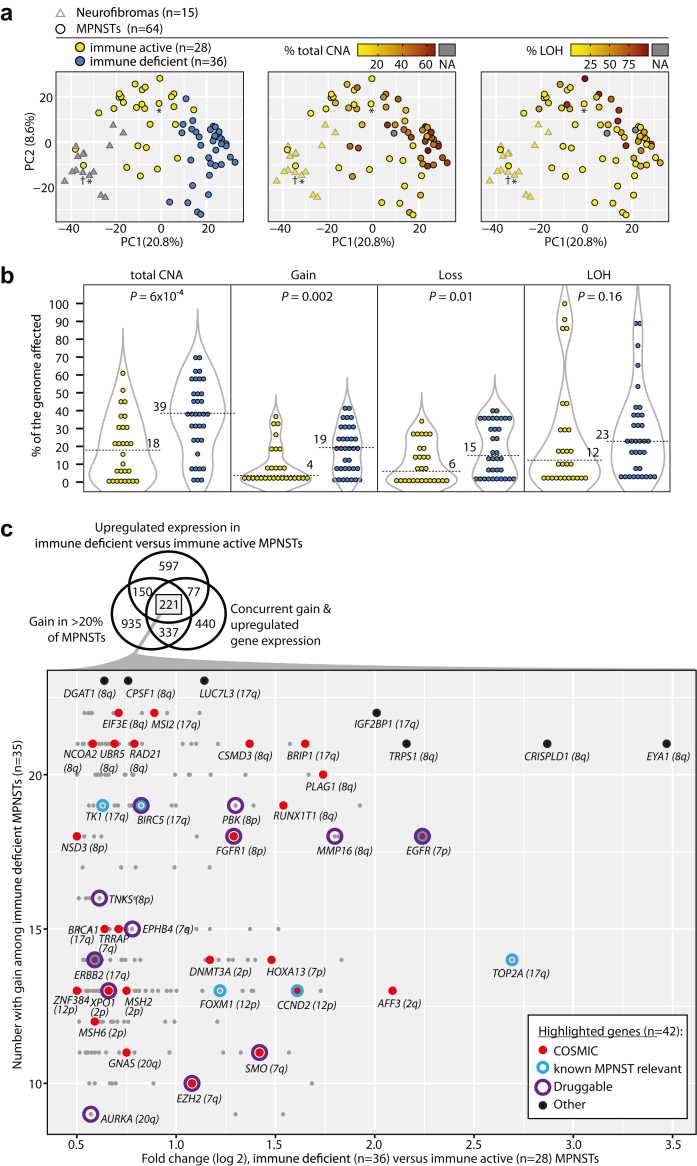
**DNA copy number aberrations according to transcriptomic MPNST subtype**. **(a)** Principal component analysis for neurofibromas and MPNSTs, coloured according to transcriptomic subtype (asterisks mark tumours from the same patient and dagger marks the plexiform neurofibroma), percentage of CNAs (gains and losses), and loss of heterozygosity (LOH). **(b)** Percentage of CNAs among immune active (yellow) and immune deficient (blue) MPNSTs. Number at dotted line is the median value. *P*-values are from Wilcoxon's test. **(c)** Venn diagram of genes with upregulated expression in immune deficient (n = 35) versus immune active MPNSTs (n = 28), with concurrent copy number gain and upregulated gene expression (investigated among 63 MPNSTs with gain versus neutral copy number), and gain in >20% of MPNSTs (investigated among 93 tumours). The scatter plot illustrates genes at the intersection of the Venn diagram (n = 221), and cancer-critical genes and top-ranked differentially expressed/aberrant genes are highlighted.

A comparison of CNA levels between the two transcriptomic MPNST subtypes revealed a higher burden of CNAs and LOH in the immune deficient MPNSTs, with the largest difference found for copy number gains (Fig. 3a and b). The genomes of immune deficient MPNSTs were also more commonly triploid than genomes of immune active tumours (OR = 5.3, *P* = 0.005 from Fisher's exact test). To investigate a potential genetic basis for the transcriptomic variation, CNAs were therefore analysed for concurrent differential expression of the target genes across MPNSTs (the tumours were grouped according to the copy number status of each gene and expression was compared in tumours with gain/loss versus neutral copy number; Supplementary Table S10). In addition to the consistent regulation of PRC2 activity at the two data levels (Fig. 2f), signalling in proliferative processes seemed to be driven by copy number gains, including targets of E2F, MYC and the G2/M checkpoint. The proportion of genes in these gene sets that was concurrently gained and upregulated was significantly higher than the median across the 50 Hallmark gene sets (*P* < 0.01 from one-sided Fisher's exact tests; Fig. 2b and Supplementary Table S9). In contrast, deregulation of immune activity did not seem to be driven by CNAs (Fig. 2b).

Differential gene expression analysis between the two transcriptomic MPNST subtypes showed that 29% of all genes with higher relative expression in immune deficient tumours were among genes with concurrent copy number gain and upregulated expression (n = 298/1045; Supplementary Table S10). Chromosome arms 8q and 17q were most frequently affected by copy number gain among the immune deficient tumours (69% and 66%, respectively), and possible target genes included *CSMD3, EIF3E, MMP16, NCOA2, RAD21,* and *UBR5* on chromosome arm 8q, and *BIRC5, BRIP1, MSI2, TK1*, and *TOP2A* on chromosome arm 17q. In addition, there were several cancer-critical genes encoding drug targets, including *EGFR* (located on chromosome arm 7p and gained in 51% of immune deficient MPNSTs), *ERBB2* (17q: 40%), *FGFR1* (8p: 51%), *SMO* (7q: 31%), and *XPO1* (2p: 37%), in addition to *EZH2* (29%); all with highest frequency of gain in immune deficient MPNSTs (OR>2.6, *P* < 0.06 from one-sided Fisher's exact test; Fig. 3c and Supplementary Table S3). Amplifications of *EGFR* and *ERBB2* were found only in immune deficient MPNSTs (n = 3 tumours with ≥15 additional copies of *EGFR* and n = 1 tumour with 9 additional copies of *ERBB2*). Notably, *ERBB2* was not differentially expressed between immune deficient MPNSTs and neurofibromas (fold change −0.44, FDR-*P* = 0.2), and differential expression between the MPNST subtypes was caused by downregulation of the gene in immune active MPNSTs compared to neurofibromas (fold change −1.03, FDR-*P* = 0.006, both from limma analysis; Supplementary Fig. S11).

Among genes with lower expression in immune deficient versus immune active MPNSTs, 20% (n = 293/1480) had concurrent loss and downregulated expression (Supplementary Table S10). This included the cancer-critical genes *ABI1, ARHGEF12, BIRC2, CBL, CYLD, JAK1, JAK2, KDM2A, NCOA4, PICALM, PTEN, RB1, SDHB, SDHD,* and *USP14* (Supplementary Fig. S12).

### Gene expression subtypes provide a framework for prognostic evaluation

The MPNST gene expression-based subtypes were independent of clinicopathological characteristics, except for a larger tumour size and enrichment with high-grade tumours in the immune deficient subtype (Table 1 and Supplementary Fig. S10d). Histopathological assessment of neighbouring tissue sections indicated a high tumour content in all samples, with no difference between immune active and immune deficient MPNSTs (*P* = 0.8, median of 95% and 100%, respectively). However, the cancer cell fraction estimated from DNA copy number data was lower in immune active than immune deficient tumours (*P* = 1 × 10^−4^, both from Wilcoxon's test, median of 0.65 and 0.75, respectively), supporting a larger microenvironment component in the immune active tumours.

**Fig. 4:**
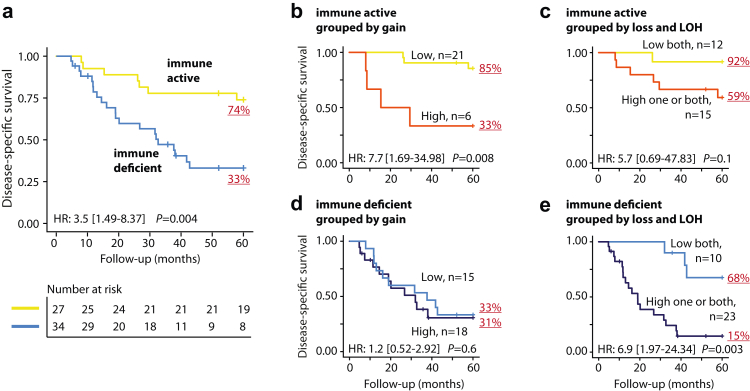
**Patient survival according to transcriptomic subtypes and DNA copy number burden**. Kaplan–Meier survival curves for patients with MPNST stratified by **(a)** transcriptomic subtypes and **(b**–**e)** transcriptomic subtypes plus copy number gain or loss/LOH. Patients were grouped according to high and low levels of CNAs using the same thresholds as for the complete MPNST series (Supplementary Fig. S8a). Hazard ratios and 95% CIs (in brackets) are from Cox regression analyses and *P*-values from Wald tests.

Consistent with the CNA associations, patients with immune deficient MPNSTs had a poorer five-year disease-specific survival than patients with immune active tumours (Fig. 4a). The MPNST subtyping framework was an independent prognostic factor in a multivariable model with clinicopathological factors (multivariable HR = 4.0, 95% CI 1.6–10.1, *P* = 0.003; Supplementary Table S8), also specifically among patients in complete remission (HR = 4.6, 95% CI 1.5–14.0, *P* = 0.007, n = 50; Supplementary Fig. S10e), and in patients with high-grade, localized (no metastasis at diagnosis) primary tumours (HR = 7.5, 95% CI 1.7–33.4, *P* = 0.008 from Wald test; Supplementary Fig. S10f). Stratification according to NF1-status indicated similar prognostic associations in NF1-associated and sporadic MPNSTs, although statistically significant in the NF1-associated only (HR = 5.3, 95% CI 1.5–19.3, *P* = 0.01 and HR = 2.3, 95% CI 0.7–7.8, *P* = 0.2, respectively, from Wald test).

**Fig. 5:**
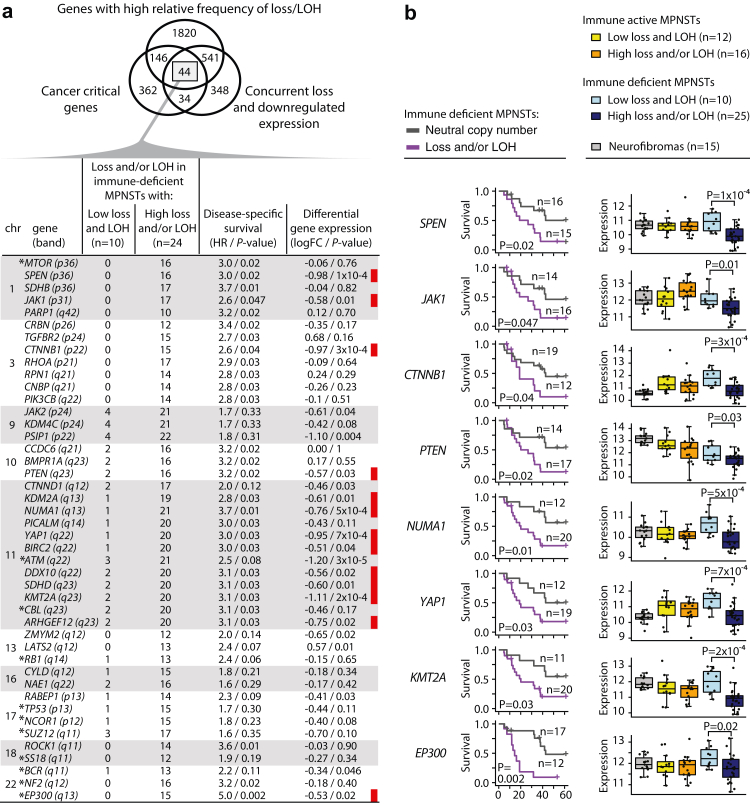
**Potential target genes in immune deficient MPNSTs with a high burden of DNA copy number loss and/or LOH**. **(a)** Venn diagram of affected genes in the subset of immune deficient MPNSTs with a particularly high frequency of loss/LOH (genes with difference in aberration frequency >40% relative to immune deficient MPNSTs with a low burden of loss and/or LOH). Thresholds to group MPNSTs according to high and low aberration levels are shown in Supplementary Fig. S8a. Cancer-critical genes with concurrent loss and downregulated expression (n = 44) were listed in the table below. The asterisk indicate genes with small-scale mutations that have previously been reported in MPNSTs.^14^^,^^15^^,^^18^^,^^44^^,^^45^ Table columns indicate from left to right the number of immune deficient MPNSTs with loss and/or LOH of each gene; associations with disease-specific survival (hazard ratios from Cox regression analysis and *P*-values from Wald tests in comparisons of immune deficient MPNSTs with neutral copy number versus loss and/or LOH of each gene); and results from differential gene expression analyses between immune deficient MPNSTs with high burden of loss and/or LOH versus low burden of both (fold change on log2-scale [logFC] and *P*-values from limma analyses). Red bars indicate genes with significant *P*-values from both survival and differential gene expression analyses and **(b)** selected genes from each chromosomal region were illustrated with Kaplan–Meier curves of disease-specific survival and box plots of gene expression in different tumour groups as indicated (log2-scale).

Immune active MPNSTs with a high burden of copy number gains had a lower relative activity of immune-related processes than immune active MPNSTs with a low burden of gains (Supplementary Fig. S13), and survival among the patients differed greatly according to the burden of copy number gains (Fig. 4b). In contrast, survival among patients with immune deficient MPNSTs differed according to the burden of copy number losses and LOH (Fig. 4d and e), and this was not associated with corresponding differences in gene expression signatures (Supplementary Fig. S13). Investigations of potential target genes among immune deficient tumours with the highest burden of copy number losses and/or LOH identified 44 cancer-critical genes with concurrent loss and downregulated expression (Fig. 5a). Several of these CNAs were associated with an inferior prognosis in the immune deficient subtype (Fig. 5b, Supplementary Tables S3 and S10).

### Markers of aggressive MPNSTs are potential actionable targets

Supervised analyses of MPNSTs from patients with a short survival time (<30 months, n = 21 poor–prognosis tumours) versus patients with a long survival time (>60 months, n = 27 good-prognosis tumours) supported the prognostic value of the transcriptomic subtypes. There was enrichment with poor-prognosis tumours in the immune deficient subtype (OR = 5.7, *P* = 0.008 from Fisher's exact test, n = 48), and deregulated gene sets between the prognostic groups and the transcriptomic subtypes were largely consistent (Fig. 2a and Supplementary Fig. S14). Poor-prognosis tumours showed upregulation of the stem cell markers *LGR5, IGF2BP1, IGF2BP3,* and *PROM1* (fold change >2), as well as the drug targets *CDK1, CDK6, EZH2, KIF11, PLK1, RRM2, TTK,* and *TYMS* (fold change >1) relative to good-prognosis MPNSTs (Supplementary Fig. S15). A comparison of good-prognosis MPNSTs versus neurofibromas showed downregulation of several genes important in Schwann cell development, myelination, and neuron function, including *CDH19, ERBB3, GAS7, NRXN1, PMP2, S100B, SCN7A,* and *SOX10* (fold change < −2; Supplementary Fig. S15).

## Discussion

This study presents an integrated genomic and transcriptomic analysis of a large series of MPNSTs, and defines a gene expression-based subtyping framework with genetic associations and prognostic relevance. Several molecular features were common to MPNSTs from patients with a poor outcome. Genomic complexity and a large genome-wide burden of copy number gains in particular, were consistent with previous studies reporting on the genomic prognostic factors of MPNSTs.^7^^,^^22^^,^^46^^,^^47^ Our study further indicated that DNA copy number gains caused increased activity of several proliferative processes, and defined a transcriptomic subtype associated with a poor survival of the patients. This subtype was strongly enriched with MPNSTs with loss of PRC2 activity, consistent with data suggesting that PRC2 loss can contribute to oncogenesis by promoting cell proliferation and growth.^14^ The marker of PRC2 inactivation, loss of H3K27me3, is known to be associated with a poor prognosis in MPNST.^48^^,^^49^

The stratification of MPNST transcriptomes (and methylomes) into two distinct groups largely corresponding to H3K27me3 status was recently suggested also by the Genomics of MPNST Consortium in a collection of MPNSTs of a similar size to ours.^16^ The sample clustering was independent of NF1-status in both studies, but there was some ambiguity with respect to the corresponding prognostic associations. Multivariable analyses suggested prognostic value independent of NF1-status, consistent with a previous study analysing H3K27me3 loss.^48^ However, subgroup analyses indicated that the prognostic associations were restricted to patients with NF1-associated MPNSTs, both according to the transcriptomic subtypes in our study and according to H3K27me3 status in the consortium study.^16^ This potential difference is not attributed to a prognostic value of NF1-status, as a meta-analysis has suggested that patients with NF1-associated and sporadic MPNSTs have similar survival outcomes.^1^ Furthermore, there are few known differences in the molecular profiles of NF1-associated and sporadic tumours.^8^^,^^24^^,^^25^^,^^50^^,^^51^ Our study did suggest that the NF1-associated were most distinct from neurofibromas at both the genomic and transcriptomic levels, and this could result in distinct molecular prognostic factors between NF1-associated and sporadic MPNSTs. However, both our study and the consortium study were limited by low patient numbers and statistical power for subgroup analyses, and larger validation studies are needed to resolve a potential interaction between H3K27me3/PRC2 loss and NF1-status with respect to patient prognosis in MPNST.

The transcriptomic subtype with PRC2 loss had a higher relative CNA burden, which is in contrast to the high CNA burden found irrespective of H3K27me3 status in the study from the Genomics of MPNST Consortium.^16^ This could be attributed to methodological differences in the scoring of PRC2 inactivation, and the lack of immunohistochemistry data for H3K27me3 is a limitation of our study. Nonetheless, the Genomics of MPNST Consortium reported several specific CNA signatures and enrichments in MPNSTs with H3K27me3 loss, as well as diploid genomes in tumours with retained H3K27me3. These data were consistent with the frequent gains of chromosome arm 8q and triploid genomes among MPNSTs with PRC2 loss in our study. *MYC* and *RAD21* have been identified as potential target genes of the 8q gain in patient-derived xenografts of MPNSTs.^52^ High *RAD21* expression might promote chromosome instability and be associated with chemotherapy resistance and a poor patient survival in breast and colorectal cancer,^53^^,^^54^ supporting the higher CNA burden and poor–prognostic associations of the MPNST subtype with frequent 8q gain and *RAD21* overexpression.

Immune activity was identified as another major discriminatory feature of the transcriptomic subtypes in our study, and according to H3K27me3 loss by the Genomics of MPNST Consortium.^16^ PRC2 loss in immune deficient MPNSTs also supported other studies showing that PRC2 inactivation promotes immune evasion, including downregulation of interferon signalling and impaired antigen presentation.^55^^,^^56^ This was mediated by reprogramming of the chromatin landscape and resulted in sensitivity to inhibition of a DNA methyltransferase and histone deacetylase in MPNST cell lines.^55^ The treatment sensitivity was further associated with upregulation of interferon pathways and MHC gene expression, thereby suggesting a potential mechanism to restore immune surveillance of these tumours. An ongoing clinical trial of MPNSTs will test the effect of the hypomethylating agent decitabine specifically in tumours with inactivation of PRC2 (ClinicalTrials.gov identifier NCT04872543). Collectively, the studies from the Genomics of MPNST Consortium and our European collaboration provide strong data for the importance of PRC2 loss in shaping the molecular biology of MPNSTs, and support the rational to therapeutically target PRC2 inactivation.

The subgroup of immune active MPNSTs appeared to evoke immune and inflammatory responses of a similar strength to that observed in benign neurofibromas, and the association with a favourable prognosis of the patients suggested anti-tumour activity of the immune response. However, immune active MPNSTs also showed immune inhibitory signals, with high relative expression of genes encoding immune checkpoint ligands. A small subgroup of the tumours appeared to have particularly strong immune evasion, suggesting vulnerability to immune checkpoint inhibition. Case reports have shown strong responses to pembrolizumab or nivolumab in PD-L1 positive MPNSTs.^57^, ^58^, ^59^, ^60^ Two ongoing early-phase trials are expected to indicate the frequency and strength of treatment responses in advanced and newly diagnosed MPNSTs (ClinicalTrials.gov identifiers NCT02691026 and NCT04465643, respectively). These studies do not incorporate patient stratification based on immune markers, but our results and results from the Genomics of MPNST Consortium^16^ suggest opportunity for immune subtyping prior to treatment. Targeting other components of the tumour immune microenvironment may also have clinical efficacy in sarcomas, including MPNSTs. An early phase trial showed therapeutic benefit from inhibition of activated M2 macrophages with a combination of CSF1R and MTOR inhibitors.^61^

There is little knowledge of the mechanisms governing the immunity of MPNSTs beyond the association with PRC2 inactivation. Small mutations typically resulting in neoantigens are not particularly frequent in MPNSTs,^14^^,^^16^^,^^18^ although cancer-specific expression of a potentially targetable cancer-testis antigen (preferentially expressed antigen in melanoma) has been shown in 66% of MPNSTs.^62^ Signalling in immune-related processes was not associated with direct targeting of the signature genes by CNAs in our study. However, the high CNA burden and frequently triploid genomes in the immune deficient subtype is consistent with data from other cancer types showing that tumour aneuploidy is associated with a poor response to immune checkpoint inhibition.^63^, ^64^, ^65^ Of particular relevance, *JAK1* and *JAK2* were identified as frequent targets of chromosomal losses and concurrent downregulated expression in the immune deficient MPNSTs. Inactivation of *JAK1/2* is a known resistance mechanism of immune checkpoint inhibition, due to subsequent loss of interferon-γ signalling.^66^ It has been suggested that frequent chromosomal losses of *JAK2* is a result of co-deletion with the tumour suppressor gene *CDKN2A* on chromosome arm 9p across cancer types.^67^ *CDKN2A* was one of few frequent targets of homozygous loss in MPNSTs in our study.^14^^,^^15^

Chromosomal loss of *CDKN2A* was also one of few recurrent events in the benign neurofibromas. Previous studies have suggested that loss of *CDKN2A* is exclusive to atypical neurofibromas and not present in benign plexiform neurofibromas.^41^ In fact, loss of *CDKN2A* has been used as a marker to suggest that atypical neurofibromas are precursor lesions of MPNSTs.^10^ The limited histopathological data available for the neurofibromas analysed in this and another study identifying loss of *CDKN2A*^14^ precluded any conclusions regarding the presence of the mutation in neurofibromas in general. Of note, the cutaneous neurofibromas were analysed as a reference of non-malignant peripheral nerve tissue in our study, and the data should be interpreted with care since the neurofibromas do not represent healthy tissue or precursors of malignant lesions. The transcriptomic MPNST subtypes were therefore analysed independently of the neurofibromas, and the interpretation of discriminatory features was not influenced by the benign lesions. Nonetheless, the comparisons of MPNST subtypes and neurofibromas supported a previous meta-analysis showing upregulation of genes related to DNA replication and cell cycle pathways in MPNSTs relative to benign tumours, as well as downregulation of genes relevant for peripheral nervous system development and immune complement activation.^68^ Our study further suggested that downregulation of immune activity was specific to the immune deficient subtype, but it is not known whether this is consistent in comparison with atypical neurofibromas. Downregulation of differentiation markers such as *SOX10* was found in the least aggressive subtype of MPNSTs relative to neurofibromas, and this has also been shown in a comparison of MPNSTs with normal Schwann cells, supporting appropriateness of the neurofibromas as a non-malignant reference.^28^

The transcriptomic classification proposed in this study appeared to capture continuous and non-discrete signals between the subtypes. Both the proliferative and immune-related gene sets, as well as the CNA burden, varied substantially within the two subtypes. Consequently, the CNA burden provided a potential for further prognostic substratification of the transcriptomic subtypes, although care should be taken in the interpretation of these results due to the low number of patients in each stratum. A continuous nature of cancer transcriptomes may be a general feature of several cancer types,^69^ and this challenges the development of subtyping frameworks, in particular of orphan malignancies for which large sample numbers are difficult to obtain. However, a subgroup of MPNSTs characterized by low or no expression of genes associated with proliferation and growth, as well as high expression of genes associated with neuroglial differentiation was also identified in a previous study,^25^ and this corresponds to the immune active MPNST subtype defined here. The consistency of transcriptomic frameworks described across studies indicates that the distinction between immune active and immune deficient/proliferative MPNSTs with PRC2 loss can indeed provide a useful starting point for more detailed characterisation and subclassification of MPNST transcriptomes.^16^^,^^25^ The increasing availability of gene expression data of MPNST samples opens up the possibility for collaborative efforts and more robust analyses in relation to clinical endpoints, and the benefit of such an approach has been illustrated in several cancer types.^70^^,^^71^

MPNST is currently regarded as a chemo-resistant cancer type, and molecular analyses to identify new cancer cell vulnerabilities are recommended.^72^ We identified candidates for targeted treatment based on consistent dysregulation of known “actionable” targets at the DNA copy number and gene expression levels. Upregulated *PLK1* was one of these targets, and we have previously identified PLK1 inhibitors to be among the most potent anticancer agents in a high-throughput drug screen of seven MPNST cell lines.^73^ Amplification and outlier expression of *EGFR*^7^^,^^19^^,^^25^^,^^74^^,^^75^ and *ERBB2* were found in a few immune deficient MPNSTs. A preclinical study has indicated a dose-dependent inhibition of proliferation of MPNST cell lines after treatment with the EGFR inhibitor erlotinib.^19^ However, a phase II clinical study of erlotinib in unresectable or metastatic MPNSTs achieved stable disease as the best response in only one of 20 patients (ClinicalTrials.gov identifier NCT00068367).^76^ The lack of molecular pre-screening prevented conclusions regarding the efficacy of a stratified treatment strategy in this trial, but targeting of the MAPK signalling pathway in MPNSTs is complicated by the frequent loss of NF1 activity,^14^^,^^18^ which suggests constitutive RAS signalling downstream of EGFR and ERBB2. In addition, genes of the ERBB family are important in normal Schwann cell differentiation, and Schwann cells may respond in different ways to signalling through these receptors.^77^^,^^78^ Therefore, downregulated expression of *ERBB2* among immune active MPNSTs may reflect disruption of the normal cellular program. Another therapeutic opportunity was suggested by concurrent DNA copy number gain and upregulated expression of *EZH2* in poor-prognosis MPNSTs.^79^ *EZH2* encodes a core component of PRC2, but might exert PRC2-independent oncogenic activity,^80^ and the EZH2 inhibitor tazemetostat has been approved by the US Food and Drug Administration for treatment of advanced epithelioid sarcomas with *EZH2* mutation. The drug is currently also evaluated against metastatic and/or treatment refractory MPNSTs (ClinicalTrials.gov identifier NCT04917042), although with no molecular pre-screening of patients. Based on our study, we hypothesize a higher response rate in molecular subgroups defined by CNAs and/or gene expression. Targeted treatment of YAP1 has also been suggested in MPNSTs.^81^ However, we show that *YAP1* has both chromosomal loss and downregulated expression in the aggressive subgroup of immune deficient MPNSTs with a high frequency of loss and LOH. Our study also validated the importance of previously identified target genes on chromosome arm 17q in MPNST, including *BIRC5, TK1,* and *TOP2A*.^22^^,^^23^^,^^68^^,^^82^^,^^83^ We have previously suggested that high expression levels of these genes and their encoded proteins represent a prognostic risk profile among patients with MPNST.^20^

In conclusion, we report an integrative molecular study of MPNSTs at the genomic and transcriptomic levels. The study is of a relatively large scale for a rare cancer type, and adds to the growing body of evidence suggesting that loss of PRC2 is a discriminatory feature of multi-omics profiles of MPNSTs and a predictor of poor patient prognosis. We propose a subtyping framework with several consistent patterns of chromosomal aberrations and expression changes. Based on the strong prognostic relevance, this framework may provide a valuable resource for future translational studies, and for potential development of biomarker-guided treatment strategies against this orphan malignancy.

## Contributors

- Planning and directing the study: M.H., A.S., R.A.L.
- Patient inclusion, collection of samples, collection and curation of clinicopathological data: B.B., K.B., T.K.G., N.M., E.v.d.B., E.P., S.S., P.P., F.M.
- Data acquisition (laboratory work) and analyses: M.H., K.C.G.B., I.A.E., M.K., O.C.L., A.S., R.A.L.
- Data interpretation: all authors.
- Verification of underlying data: M.H., K.C.G.B.
- Drafting of manuscript: M.H., K.C.G.B., A.S., R.A.L.
- Revisions and final approval of the manuscript: all authors.
- Coordinating revisions, finalization of the manuscript and responsibility for the decision to submit: M.H., A.S., R.A.L.
- Study supervision: A.S., R.A.L.

## Data sharing statement

Gene expression data from Human Transcriptome 2.0 Arrays have been deposited to NCBI's Gene Expression Omnibus and can be accessed from GSE241224. In accordance with Norwegian legislation and the ethical approval of the study by the Regional Committee for Medical and Health Research Ethics, South East Norway, raw data from Human SNP Arrays 6.0 are considered patient identifiable and subject to secure storage regulations. Data can currently not be deposited to public repositories, but will be made available upon reasonable request to the corresponding author, and this will require formalization of a data transfer agreement.

## Declaration of interests

Authors declare that they have no competing interests related to this study. Payments/honoraria from companies for written or oral presentations or lectures are reported by K.B. (Novartis) and O.C.L (Nykode) and for consulting by O.C.L. (Novartis Norway). K.B. also reports participation on a Data Safety Monitoring Board or Advisory Board for GSK, Bayer, NEC OncoImmunity, and Incyte.
