# Supplementary material for: Transcriptomic subtyping of malignant peripheral nerve sheath tumours highlights immune signatures, genomic profiles, patient survival and therapeutic targets
Source: eBioMedicine. 2023 Oct 12;97:104829. doi: 10.1016/j.ebiom.2023.104829 (PMC10585232; doi:10.1016/j.ebiom.2023.104829)
Supplement: Supplementary Methods [file mmc2.docx]

## Supplementary Methods

### High resolution DNA copy number analyses

After scanning of the genome-wide Human SNP Array 6.0 (Thermo Fisher Scientific, Waltham, MA, USA), the CEL files were preprocessed according to the PennCNV protocol[^1^](#_ENREF_1) adapted for Affymetrix genotyping arrays,[^2^](#_ENREF_2) and probe filtering was performed as described.[^3^](#_ENREF_3) HapMap samples (n=270 individuals)[^4^](#_ENREF_4) were used as reference for quantile normalization and calculation of Log R Ratio (LRR) and B Allele Frequency (BAF). Probes targeting autosomes were kept for further analyses. Single-sample winsorization and segmentation of LRR values were performed by the PCF algorithm implemented in the Bioconductor R package copynumber (v1.12.0; penalty parameter, γ=100; minimum number of probes per segment, kmin=5).[^5^](#_ENREF_5) Regions with CNAs, including loss (LRR estimates ≤-0.15) and gain (LRR estimates ≥0.15), were identified from sample-wise median centred data, and target genes were identified with the GISTIC algorithm (v2.0.23).[^6^](#_ENREF_6)^,^[^7^](#_ENREF_7) Peak regions containing ≤30 genes were considered focal, and a q-value below 0.25 was regarded as significant. Genome and chromosome plots were made using the copynumber package (v1.16.0).[^5^](#_ENREF_5)

LRR and BAF values were used as input to the ASCAT algorithm (v2.3; penalty parameter=50),[^8^](#_ENREF_8) providing allele specific copy numbers for 91 MPNSTs, 28 neurofibromas, and 18 white blood cell samples (two MPNSTs, where a ploidy estimate could not be decided, failed the algorithm). ASCAT segmented data were used to identify regions and genes with amplification and high-level amplification (gain of ≥5 and ≥10 copies, respectively, relative to the median genome-wide copy number per sample), homozygous loss (estimates of both A and B allele was 0), and loss of heterozygosity (LOH, estimates of either A or B allele was 0). Notably, homozygous regions in MPNSTs were considered events of tumour-associated LOH, although patient-matched germline references were not available. However, a larger proportion of the genome of MPNSTs was in a homozygous state (median=19.4%) compared to the control set of white blood cell samples (median=0.23%, *P*=1x10^-9^, Wilcoxon test) and neurofibromas (median=0.44%, *P*=2x10^-11^). In addition, the homozygous regions in MPNSTs were considerably longer (average length of 18.7 Mb) than in the blood cells (3.1 Mb, *P*=4x10^-7^, Fisher’s exact test) and neurofibromas (3.7 Mb, *P*=5x10^-6^).

The percentage of the genome with aberrant copy number or LOH was calculated from the number of aberrant bases out of the total number of bases with copy number/LOH estimates available.

Gene-level copy number estimates were retrieved as described in Supplementary table 3, and 18,091 protein-coding autosomal genes were successfully mapped to PCF and ASCAT segmented data.

### Gene expression profiling

Differential gene expression analysis between sample groups was performed with the R package limma (v3.32.10),[^9^](#_ENREF_9) and the criteria for calling significantly differentially expressed genes was set to an expression variance across MPNSTs of >0.3, *P*-values adjusted for false discovery rate (FDR-*P)*<0.05, and log2 fold change >|0.5|. Unsupervised gene expression-based classification was performed using the R package NMF (v0.22.0, functions: nmfEstimateRank and nmf with default settings except for nrun=100), with 2,388 genes as input (gene expression variance >1 among MPNSTs). Principal component analysis was performed by using the function prcomp (with scaling and centering) from the R stats package, including the same genes as in unsupervised classification (n=2,388).

## References

1 Wang K, Li M, Hadley D, Liu R, Glessner J, Grant SF, et al. PennCNV: an integrated hidden Markov model designed for high-resolution copy number variation detection in whole-genome SNP genotyping data. Genome Res. 2007;17(11):1665-74.

2 Webpage. PennCNV-Affy. Protocol for CNV detection in Affymetrix SNP arrays. Available at: http://www.openbioinformatics.org/penncnv/penncnv_tutorial_affy_gw6.html. Accessed Mar 20 2017.

3 Sveen A, Loes IM, Alagaratnam S, Nilsen G, Holand M, Lingjaerde OC, et al. Intra-patient Inter-metastatic Genetic Heterogeneity in Colorectal Cancer as a Key Determinant of Survival after Curative Liver Resection. PLoS genetics. 2016;12(7):e1006225.

4 McCarroll SA, Kuruvilla FG, Korn JM, Cawley S, Nemesh J, Wysoker A, et al. Integrated detection and population-genetic analysis of SNPs and copy number variation. Nat Genet. 2008;40(10):1166-74.

5 Nilsen G, Liestol K, Van Loo P, Moen Vollan HK, Eide MB, Rueda OM, et al. Copynumber: Efficient algorithms for single- and multi-track copy number segmentation. BMC Genomics. 2012;13:591.

6 Beroukhim R, Getz G, Nghiemphu L, Barretina J, Hsueh T, Linhart D, et al. Assessing the significance of chromosomal aberrations in cancer: methodology and application to glioma. Proc Natl Acad Sci U S A. 2007;104(50):20007-12.

7 Mermel CH, Schumacher SE, Hill B, Meyerson ML, Beroukhim R, Getz G. GISTIC2.0 facilitates sensitive and confident localization of the targets of focal somatic copy-number alteration in human cancers. Genome Biol. 2011;12(4):R41.

8 van Loo P, Nordgard SH, Lingjaerde OC, Russnes HG, Rye IH, Sun W, et al. Allele-specific copy number analysis of tumors. Proc Natl Acad Sci U S A. 2010;107(39):16910-5.

9 Ritchie ME, Phipson B, Wu D, Hu Y, Law CW, Shi W, et al. limma powers differential expression analyses for RNA-sequencing and microarray studies. Nucleic Acids Res. 2015;43(7):e47.
