# Supplementary material for: Transcriptomic subtyping of malignant peripheral nerve sheath tumours highlights immune signatures, genomic profiles, patient survival and therapeutic targets
Source: eBioMedicine. 2023 Oct 12;97:104829. doi: 10.1016/j.ebiom.2023.104829 (PMC10585232; doi:10.1016/j.ebiom.2023.104829)
Supplement: Supplementary Figures [file mmc3.docx]

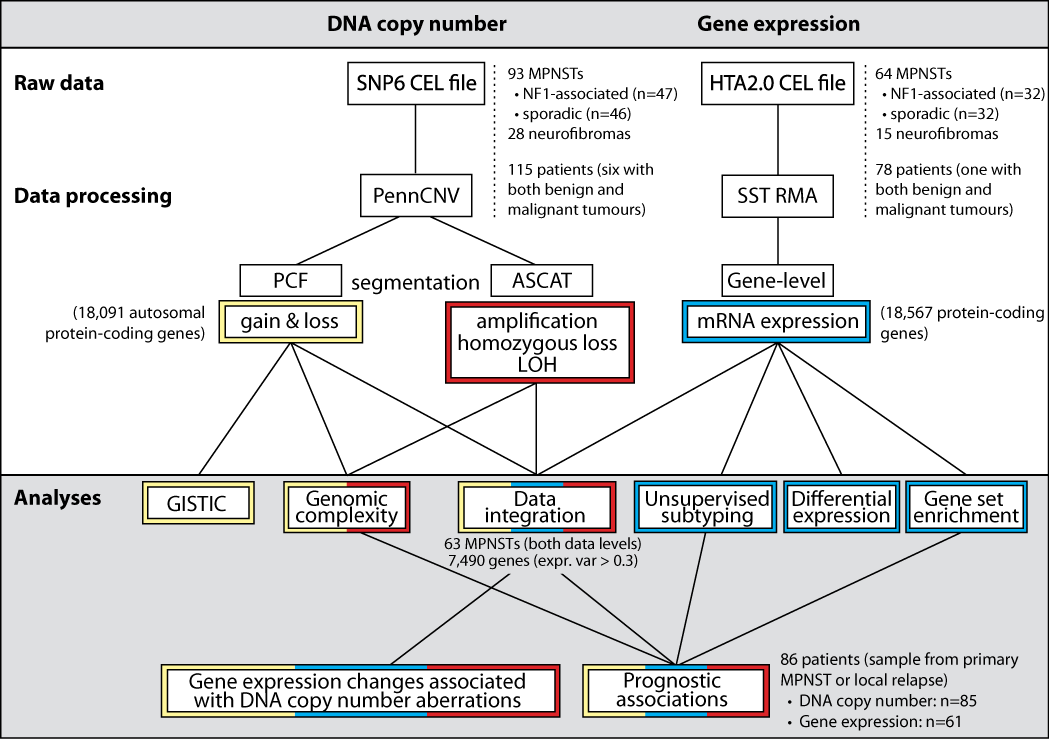


# Supplementary Fig. 1. Data analysis overview

Overview of methods and the number of genes and patients/samples included for analyses of DNA copy numbers, gene expression and clinicopathological associations.


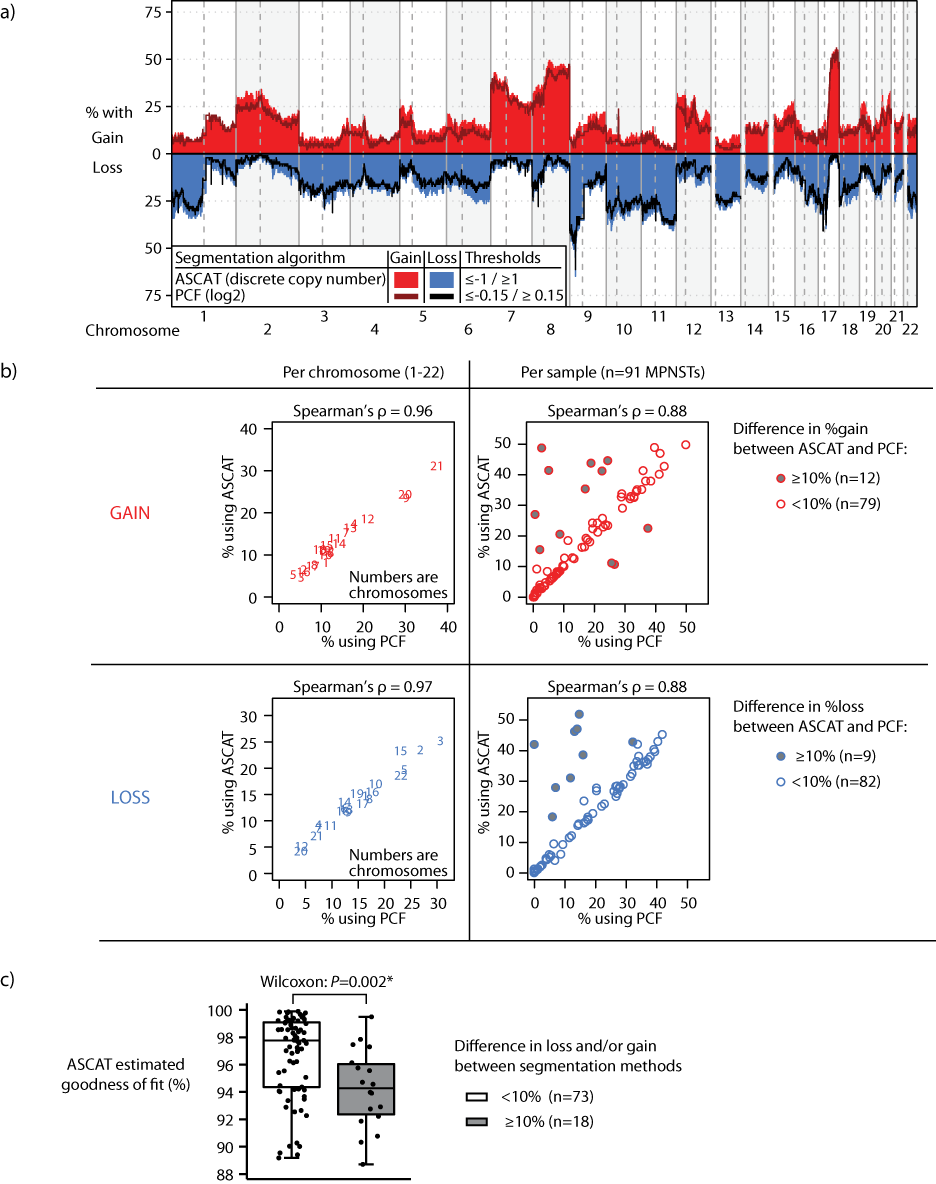


# Supplementary Fig. 2. Copy number aberration calling according to the ASCAT and PCF segmentation algorithms

**(a)** Frequency of copy number aberrations in malignant peripheral nerve sheath tumours (MPNSTs) compared for the segmentation methods ASCAT (n=91) and PCF (n=93). **(b)** Correlation between the % of gain and loss called by ASCAT and PCF across the chromosomes and per tumour sample. **(c)** ASCAT estimated goodness of fit in MPNSTs with low and high difference in copy number estimates between the methods (tumours with ≥10% difference in the genome wide percentage of losses or gains between the two segmentation methods had a significantly lower ASCAT estimated goodness of fit, indicating that the ASCAT algorithm was less certain in these cases (van Loo et al.,Proc Natl Acad Sci U S A. 2010;107(39):16910-16915)).


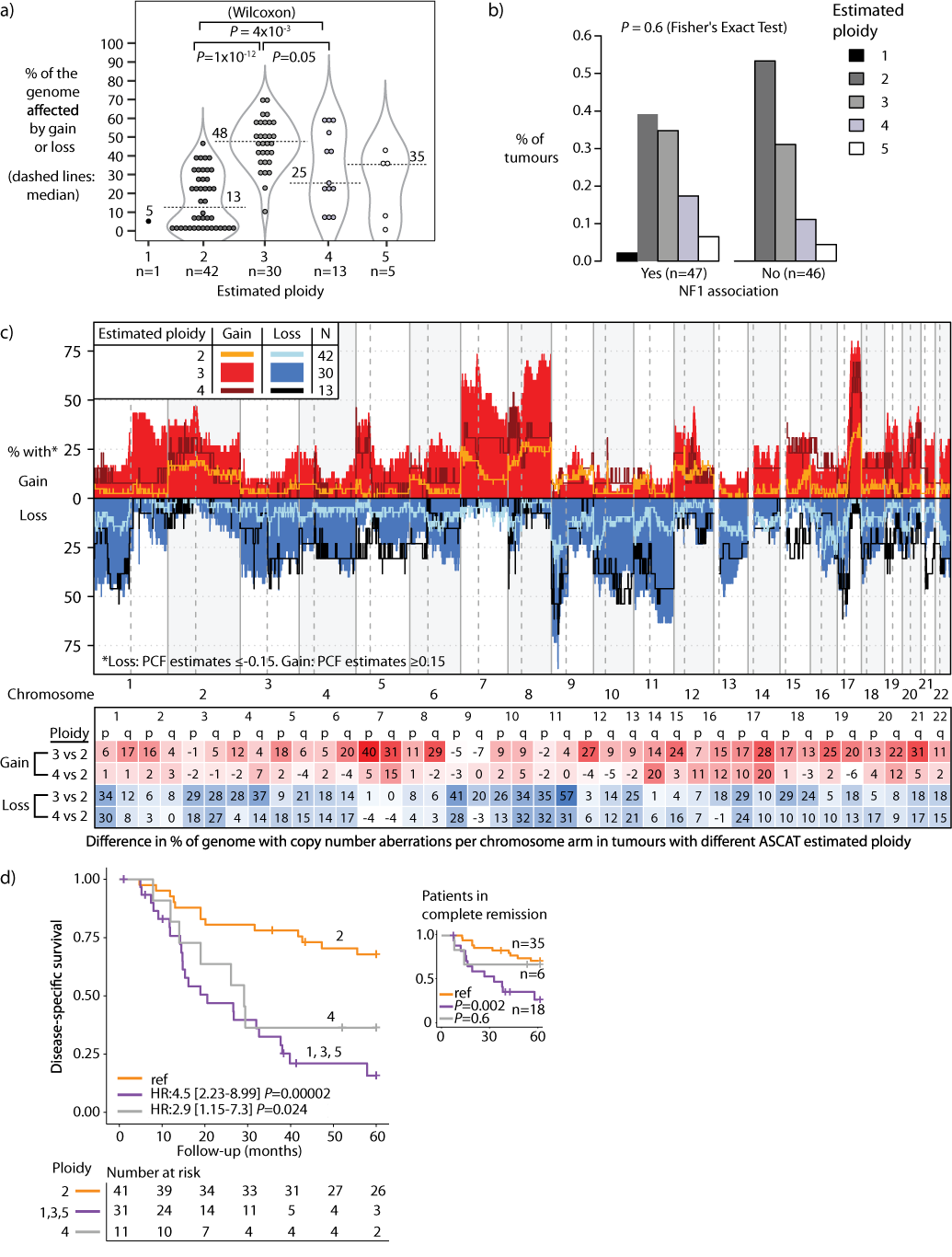


# Supplementary Fig. 3. Copy number aberrations in MPNSTs grouped by ploidy estimated by ASCAT

**(a)** Burden of genome-wide copy number aberrations per tumour sample grouped according to estimated ploidy. **(b)** Difference in ploidy in NF1-associated and sporadic MPNSTs. **(c)** Frequency of copy number aberrations in MPNSTs grouped according to estimated ploidy. **(d)** Kaplan-Meier survival curves for patients grouped according to tumour ploidy. Left, all patients; right, patients in complete remission. Patients in complete remission had wide or marginal surgical margins after removal of the primary tumour and no metastasis at diagnosis.


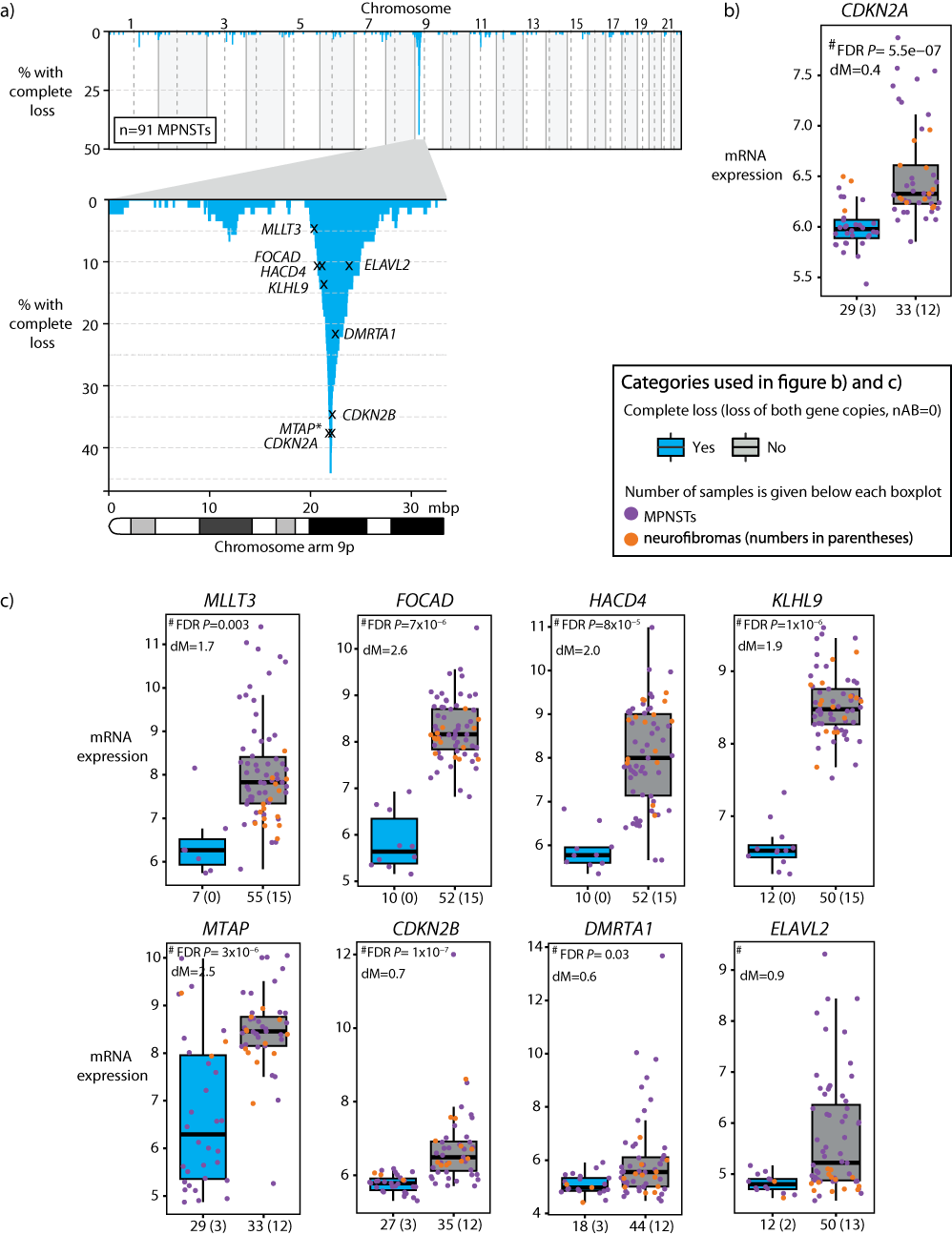


# Supplementary Fig. 4. Complete (homozygous) loss of chromosome band 9p21.3 and the effect on gene expression levels

**(a)** Frequency of complete loss across the genome of MPNSTs (n=91), highlighting the peak at 9p. **(b)** Expression of *CDKN2A* in MPNSTs and neurofibromas. **(c)** Additional genes on 9p21.3 with significantly downregulated expression associated with complete gene loss. The *MTAP* transcript stretches across *CDKN2A* (genes are mapped according to the transcript database TxDb.Hsapiens.UCSC.hg19.knownGene in R, which uses the smallest start and the largest end position for any available transcript). The hashtags indicate the FDR-adjusted *P*-values estimated by Wilcoxon rank-sum test of MPNSTs with versus without complete loss of the gene (adjusted for the 44 genes with ≥3 samples with complete loss). dM indicate the difference in median expression.


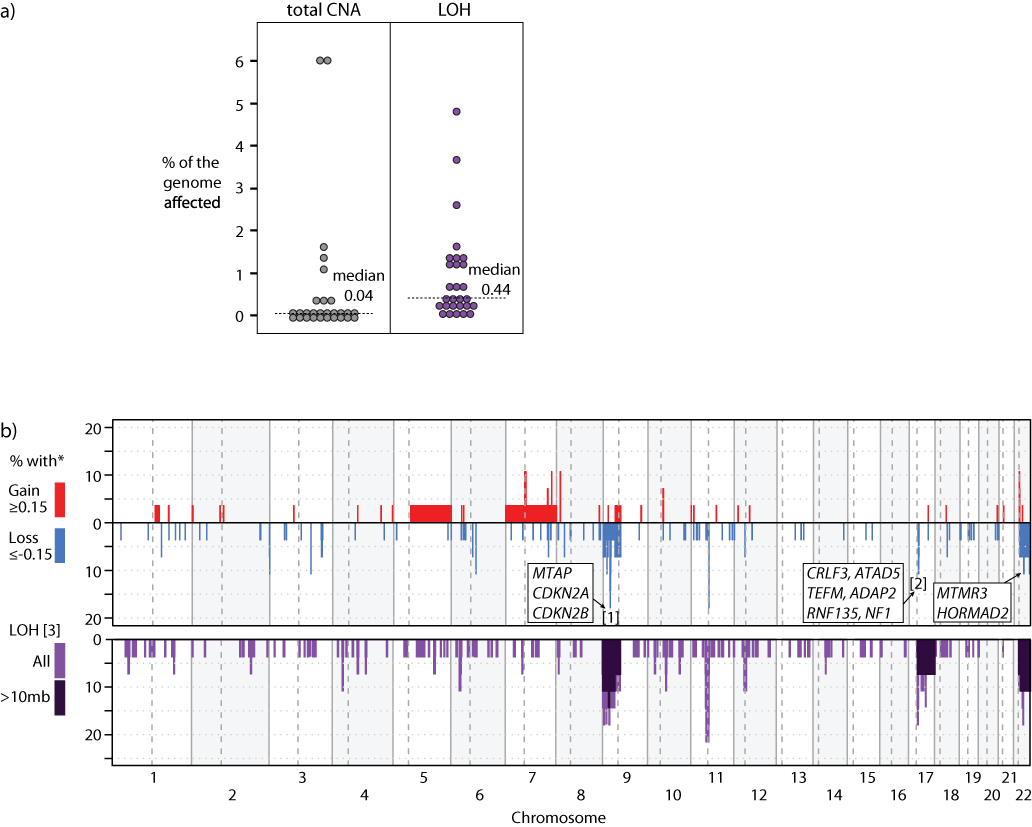


# Supplementary Fig. 5. DNA copy number aberrations in neurofibromas (n=28)

**(a)** Burden of genome-wide copy number aberrations (total CNA includes gains and losses) and loss of heterozygosity (LOH) per neurofibroma. **(b)** Frequency of copy number aberrations in neurofibromas. Gene names are given for regions with aberrations in ≥3 neurofibromas regarded as significant targets in cancer. [1] Homozygous loss in 4 of 5 (ASCAT nAB = 0). [2] The region was lost in DNA from white blood cells in one patient (not from the same patient as the analysed neurofibromas). Three neurofibromas had loss of the genes listed, two of which also had loss of the upstream gene *SUZ12*. [3] Homozygous regions, defined as regions where nA or nB is absent, can be due to inheritance or LOH. Segments longer than 10 mega bases are shown in darker purple (longer stretches of homozygosity is an indicator of non-inherited events).


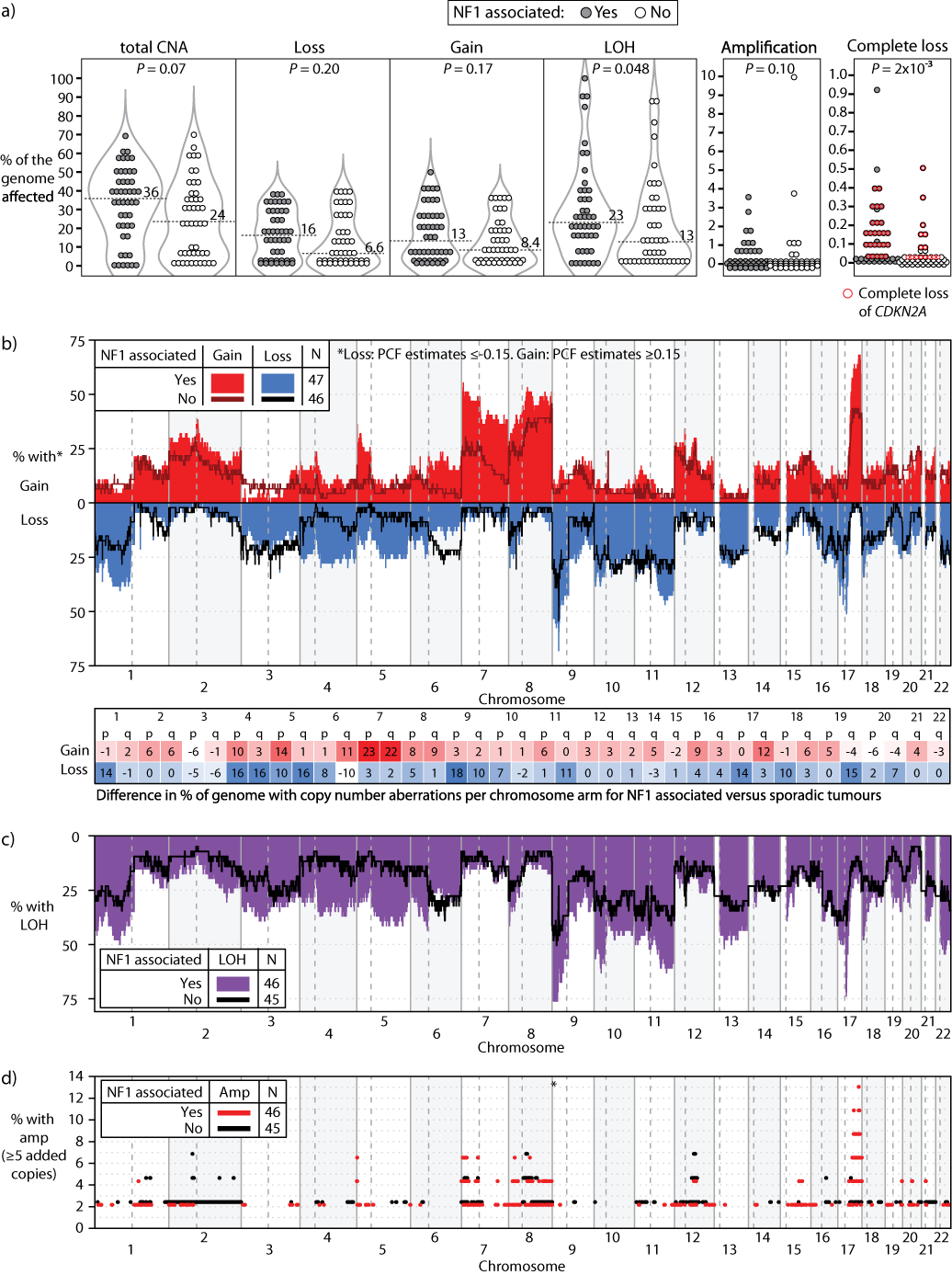


# Supplementary Fig. 6. Genome-wide DNA copy number aberrations in NF1-associated and sporadic MPNSTs

**(a)** Burden of genome-wide copy number aberrations (total CNA includes gains and losses) and loss of heterozygosity (LOH) of NF1-associated and sporadic tumours. Amplifications were called at regions with gain of ≥ 5 copies relative to the median genome-wide copy number status. *P*-values were from Wilcoxon rank-sum test and median values were indicated at the dashed lines. **(b)** Frequency of copy number aberrations across the genome. **(c)** Frequency of LOH across the genome. **(d)** Frequency of amplifications across the genome (to improve the visibility, lines were not connected and red and black dots were jittered).

Supplementary Fig. 7, page 1 of 6


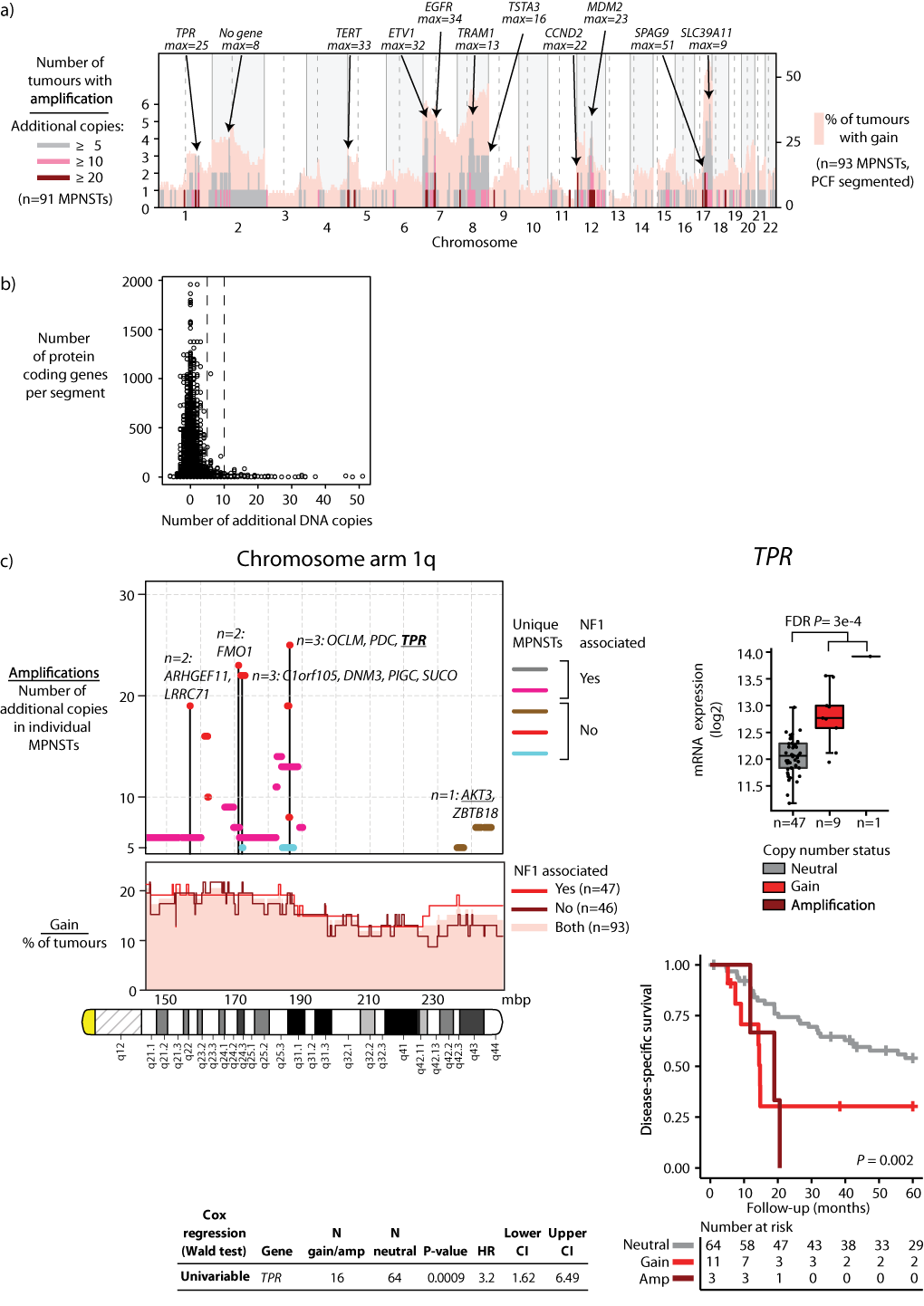


Supplementary Fig. 7, page 2 of 6


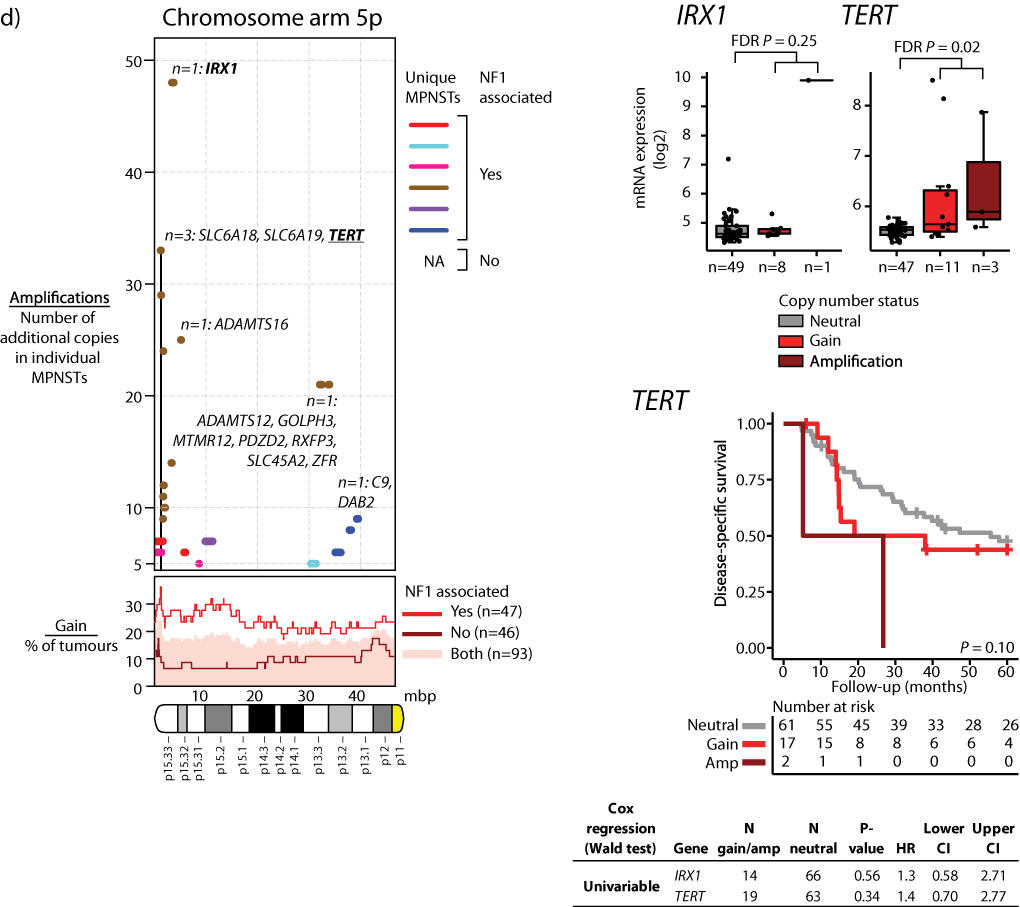


Supplementary Fig. 7, page 3 of 6


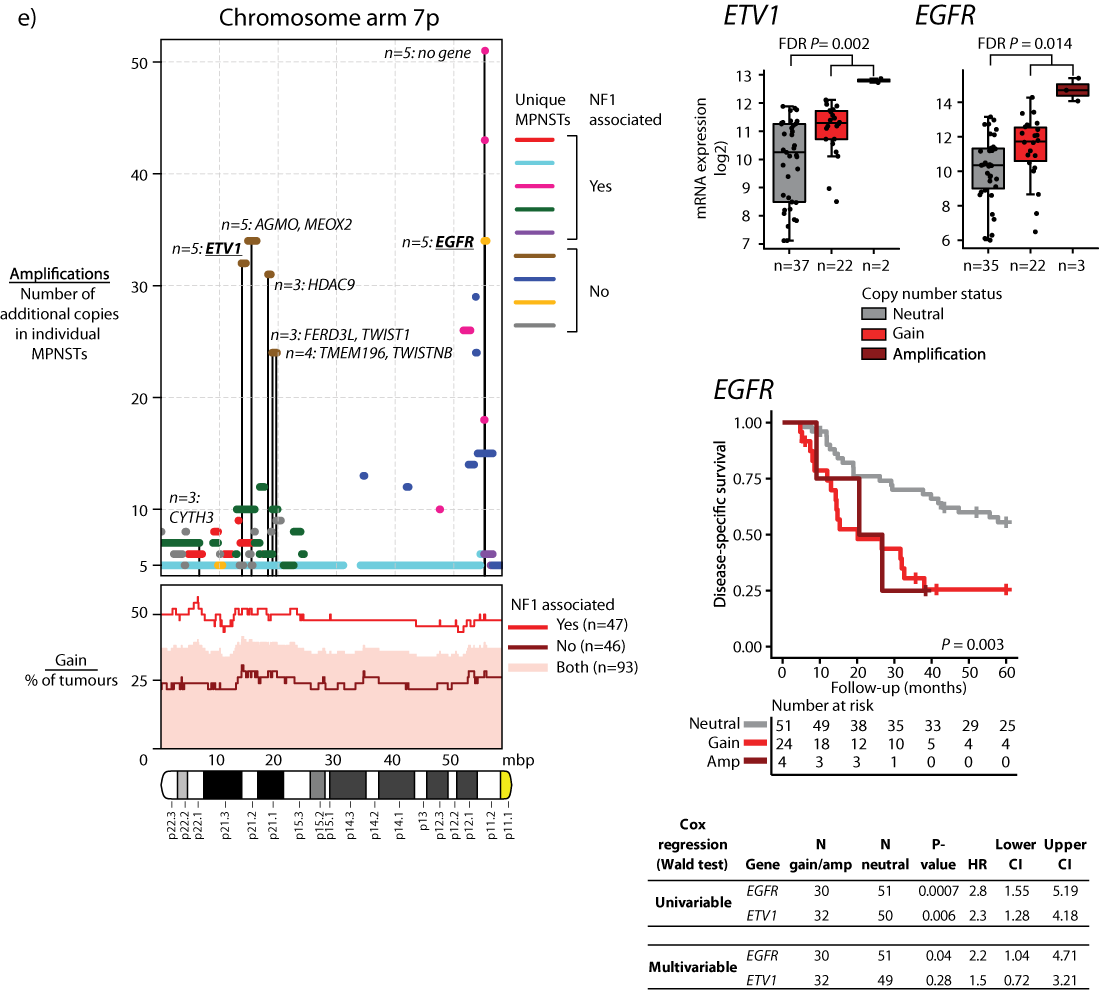


Supplementary Fig. 7, page 4 of 6


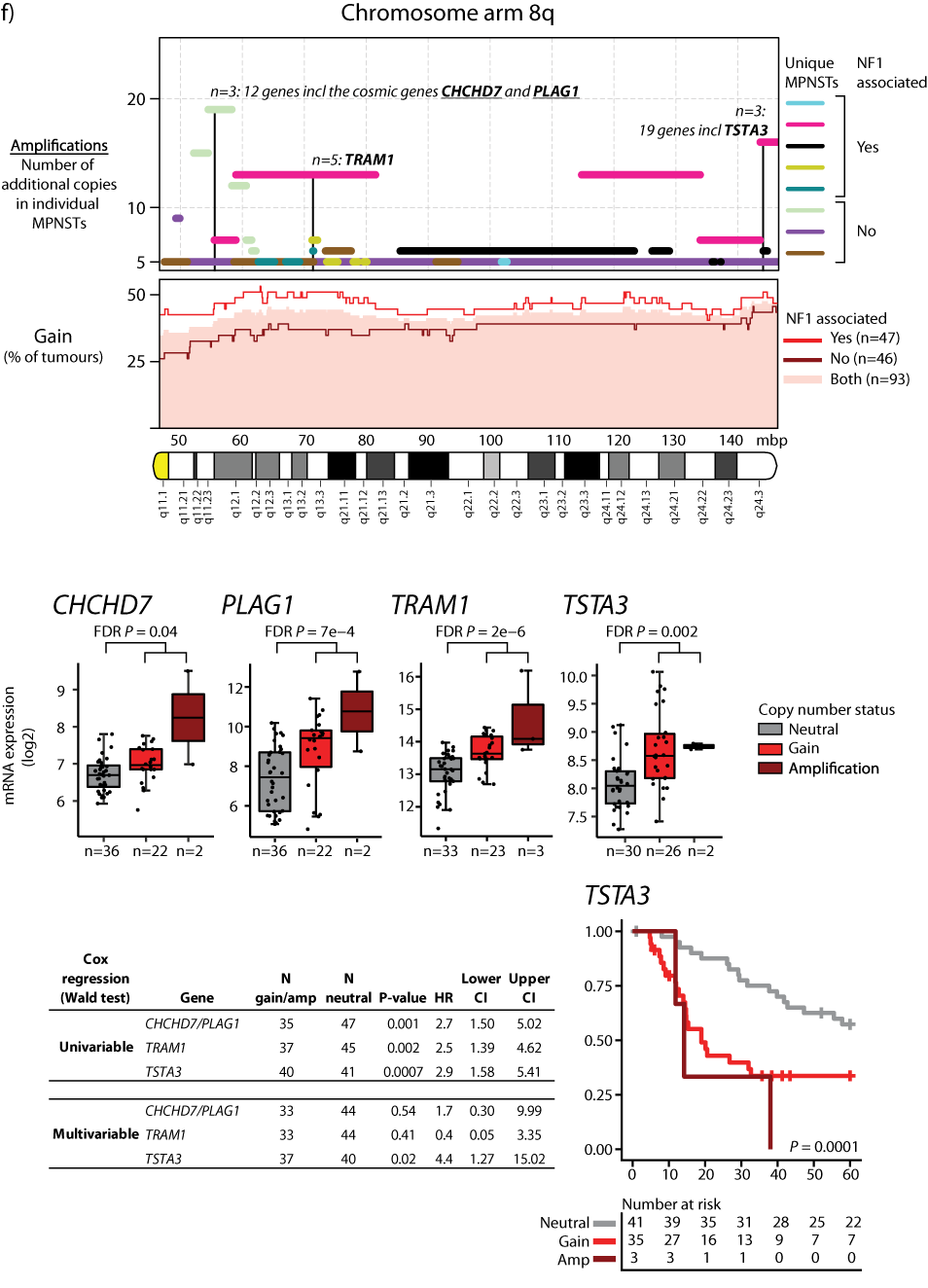


Supplementary Fig. 7, page 5 of 6


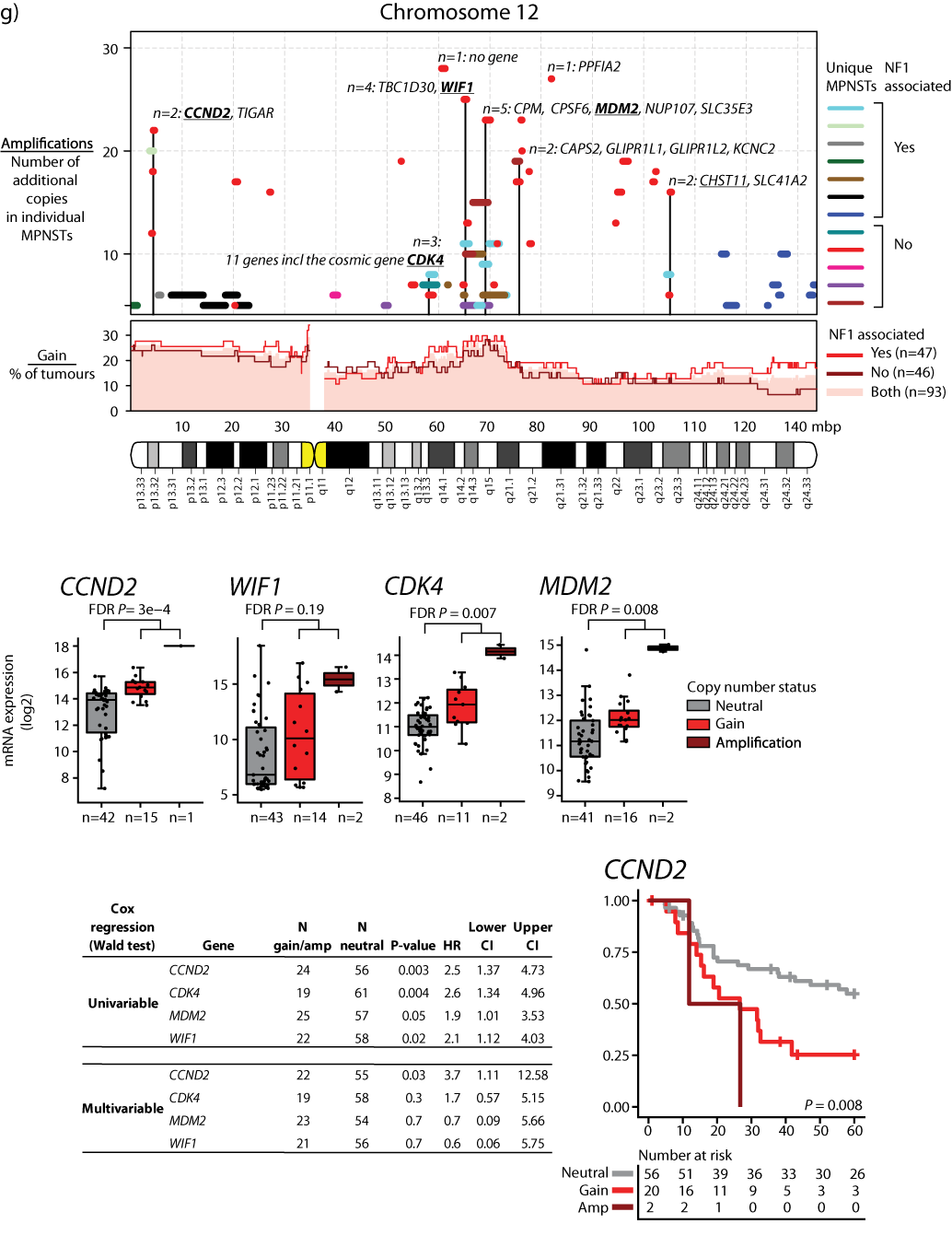


Supplementary Fig. 7, page 6 of 6


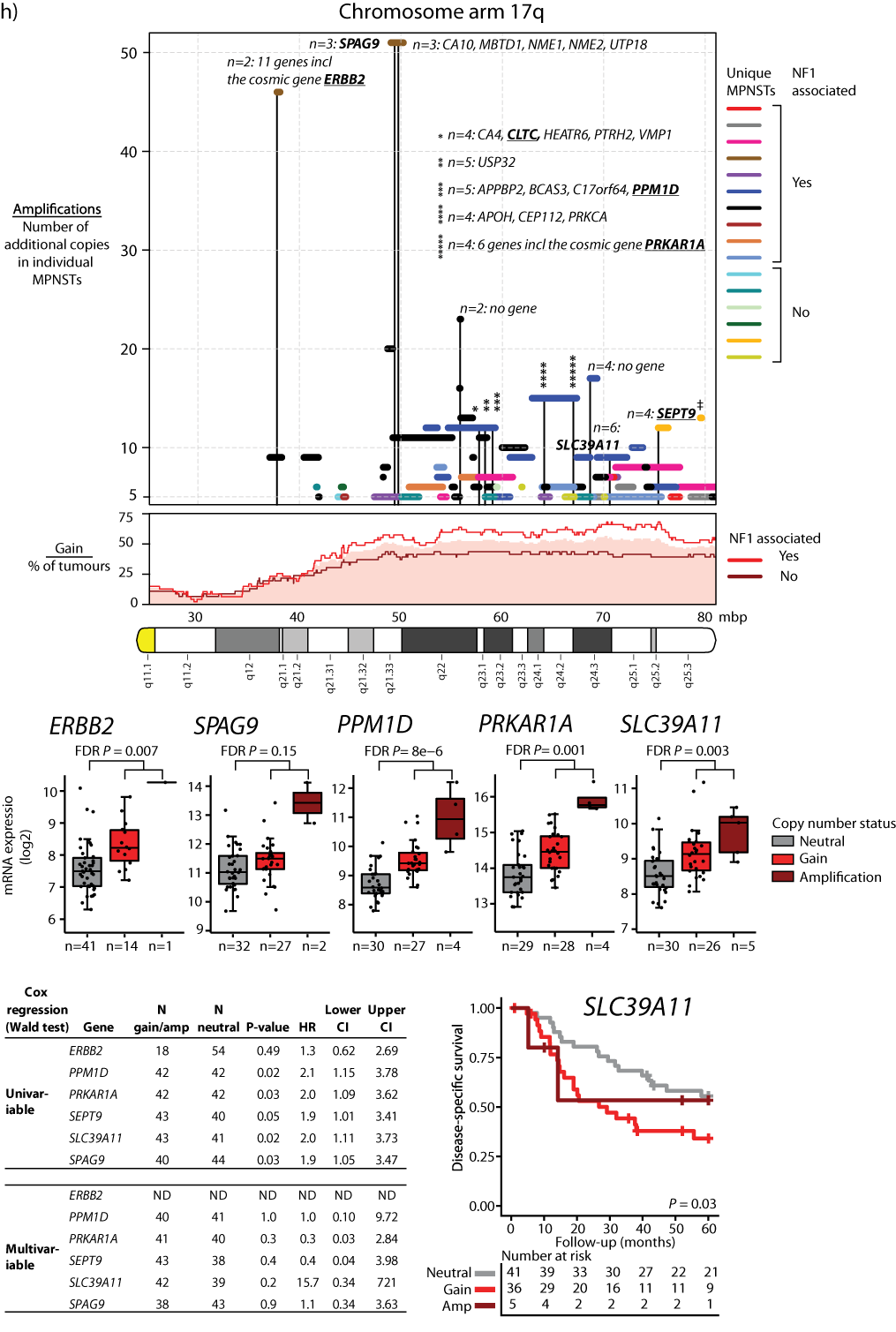


# Supplementary Fig. 7. Amplifications in MPNSTs

**(a)** Amplifications (left axis) and gains (right axis) across the MPNST genome. **(b)** Number of protein-coding genes per ASCAT segment plotted according the number of DNA copies (additional copies relative to the median genome-wide copy number status). **(c-h)** Chromosome arms 1q, 5p, 7p, 8q, 12p, 12q, and 17q had protein-coding genes with recurrent high-amplitude amplifications (5 additional copies in ≥ 3 MPNSTs and 10 additional copies in one of these, or 10 additional copies in ≥ 2 MPNSTs). All MPNSTs with ≥ 5 additional copies were included for plotting. Segments with the most frequent and/or the most extreme amplifications are labelled with vertical lines and annotated with genes. COSMIC genes are underlined and genes of particular interest are marked in bold (either the only gene in the amplified peak or genes included in COSMIC). Boxplots show gene expression according to copy number status of the genes of interest (*P*-values were from Wilcoxon rank-sum test of tumours with gain/amplification versus neutral copy number), and Kaplan-Meier survival curves are for the copy number status of the same genes (*P*-values from Breslow test for the linear trend of neutral copy number, gain and amplification). The corresponding chromosome or chromosome arm were also analyzed in Cox proportional hazards models with *P*-values from Wald test and results are shown in table format (discrepant sample numbers between the different types of survival analyses were due to failure of the ASCAT algorithm to score and call amplifications in some samples).


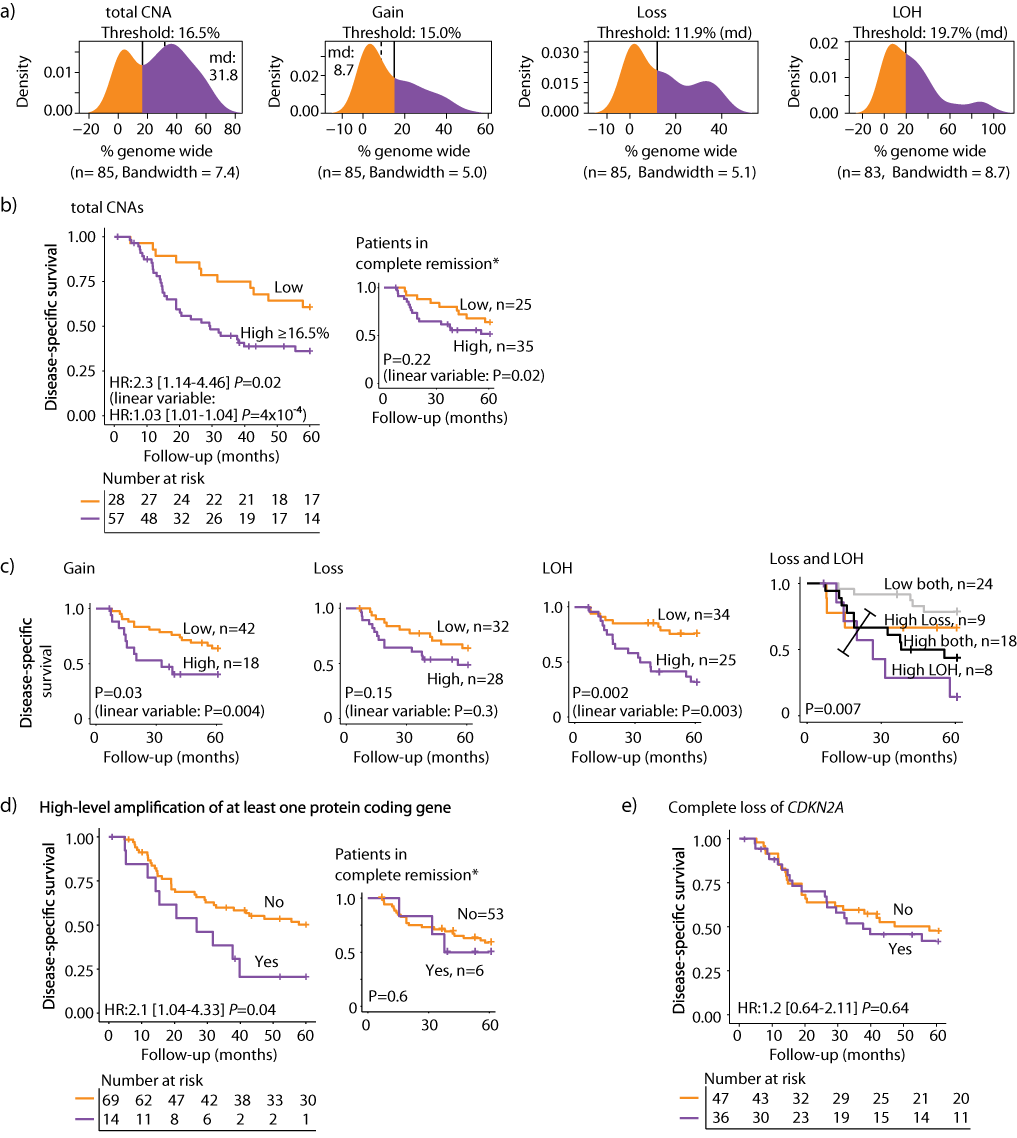


# Supplementary Fig. 8. Tumour burden of genome-wide DNA copy number aberrations is associated with prognosis in MPNST

**(a)** Density curves and thresholds for dichotomization of patients included in survival analyses according to the percentage of the genome affected by CNAs (total CNAs include gains and losses). Vertical lines are drawn at the thresholds used for dichotomisation, and groups with low and high burden of aberrations are shown in orange and purple, respectively. For total CNA and gain, the median (md) is shown by a dotted line, while for loss and LOH the threshold used was the median value. **(b)** Kaplan-Meier survival curves for patients with low and high percentage of total CNAs across the genome, shown for all patients (left) and for patients in complete remission (right). Patients in complete remission had wide or marginal surgical margins after removal of the primary tumour and no metastasis at diagnosis (same threshold for dichotomization). **(c)** Kaplan-Meier survival curves for patients in complete remission according to burden of gain, loss and/or LOH. **(d)** Kaplan-Meier survival curves for patients with (Yes) or without (No) high-level amplification (gain of ≥ 10 additional copies) of at least one protein-coding gene. **(e)** Kaplan-Meier survival curves for patients with (Yes) or without (No) complete loss of *CDKN2A*.


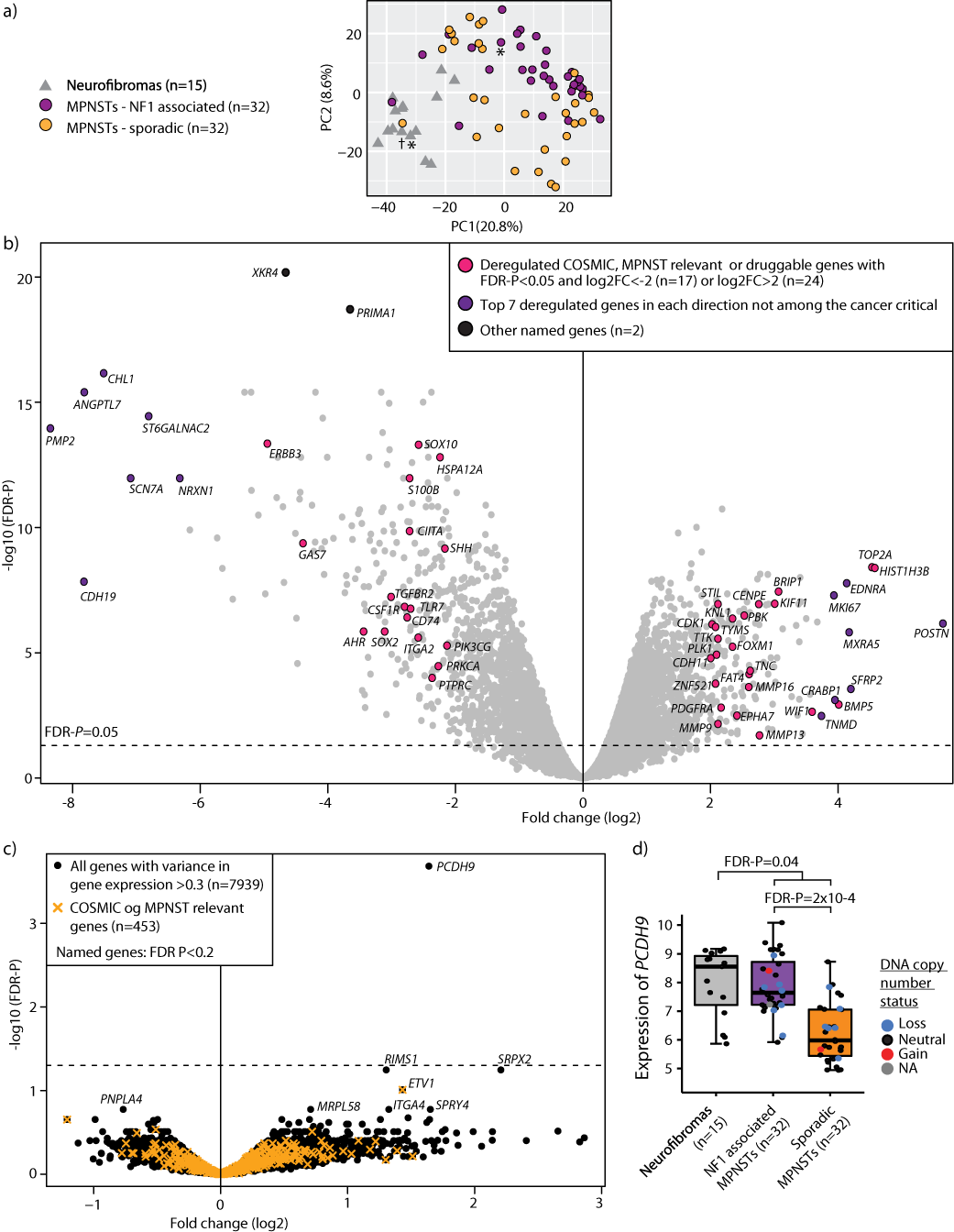


# Supplementary Fig. 9. Gene expression in neurofibromas versus MPNSTs and according to NF1-status

**(a)** Principal components analysis of genes with high variance in gene expression among MPNSTs (n=2388 genes with variance > 1). Asterisks indicate tumours from the same patient had dagger indicates the plexiform neurofibroma. **(b)** Volcano plot of differential gene expression between MPNSTs (n=64) and neurofibromas (n=15). **(c)** Volcano plot of differential gene expression between NF1-associated and sporadic MPNSTs. Both volcano plots included genes with expression variance > 0.3 (n=7939). **(d)** Expression of *PCDH9* in benign neurofibromas, NF-1 associated MPNSTs and sporadic MPNSTs. The gene copy number status is indicated in colour.


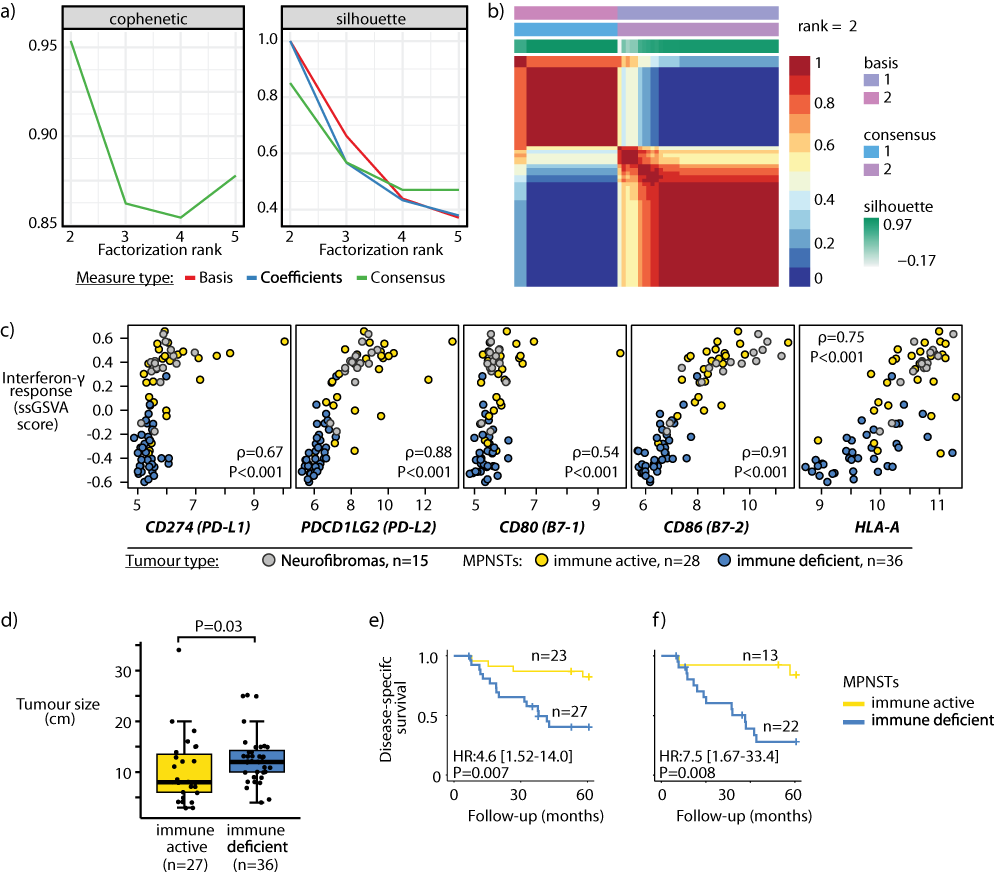


# Supplementary Fig. 10. Characteristics of the unsupervised gene expression-based subtypes of MPNSTs

**(a-b)** Metrics to evaluate the optimal number of sample clusters from subtyping of MPNSTs (n=64) by non-negative matrix factorization. **(c)** Associations between the single-sample enrichment score (ssGSVA) of the interferon-γ gene set and expression levels of selected immune-related genes. The ssGSVA scores were estimated with gene set variation analysis implemented in the R package GSVA. **(d)** Difference in tumour size between the MPNST subtypes. Survival for **(e)** patients in complete remission and **(f)** patients with high-grade, localized (no metastasis) primary tumours according to the MPNST subtypes.


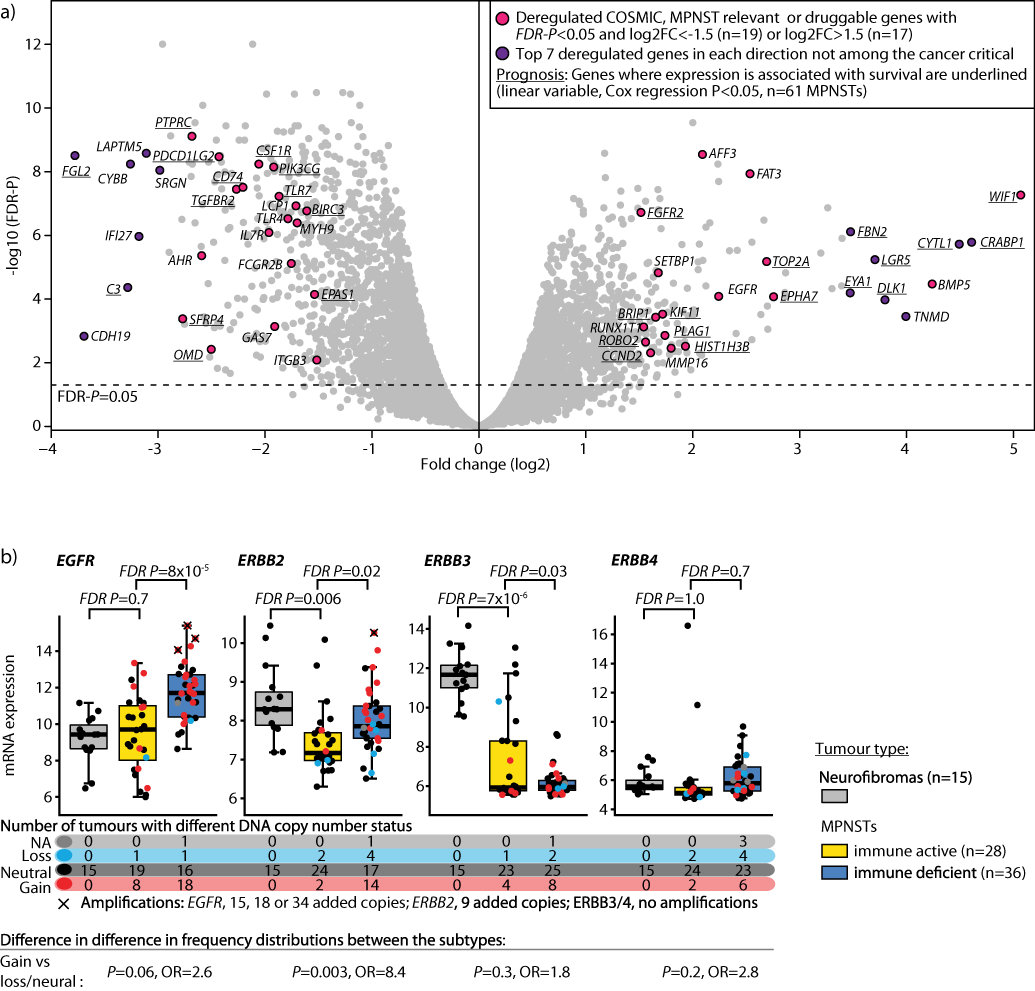


# Supplementary Fig. 11. Differential gene expression of the immune active and immune deficient MPNSTs

**(a)** Volcano plot of differential gene expression of immune deficient versus immune active MPNSTs, including genes with expression variance > 0.3 (n=7939). **(b)** Expression levels of genes of the ERBB family. *P*-values and odds ratios (ORs) are from one-sided Fisher’s exact tests of the difference in frequency distributions of gain vs loss/neural between the subtypes. The copy number status of each gene is indicated.


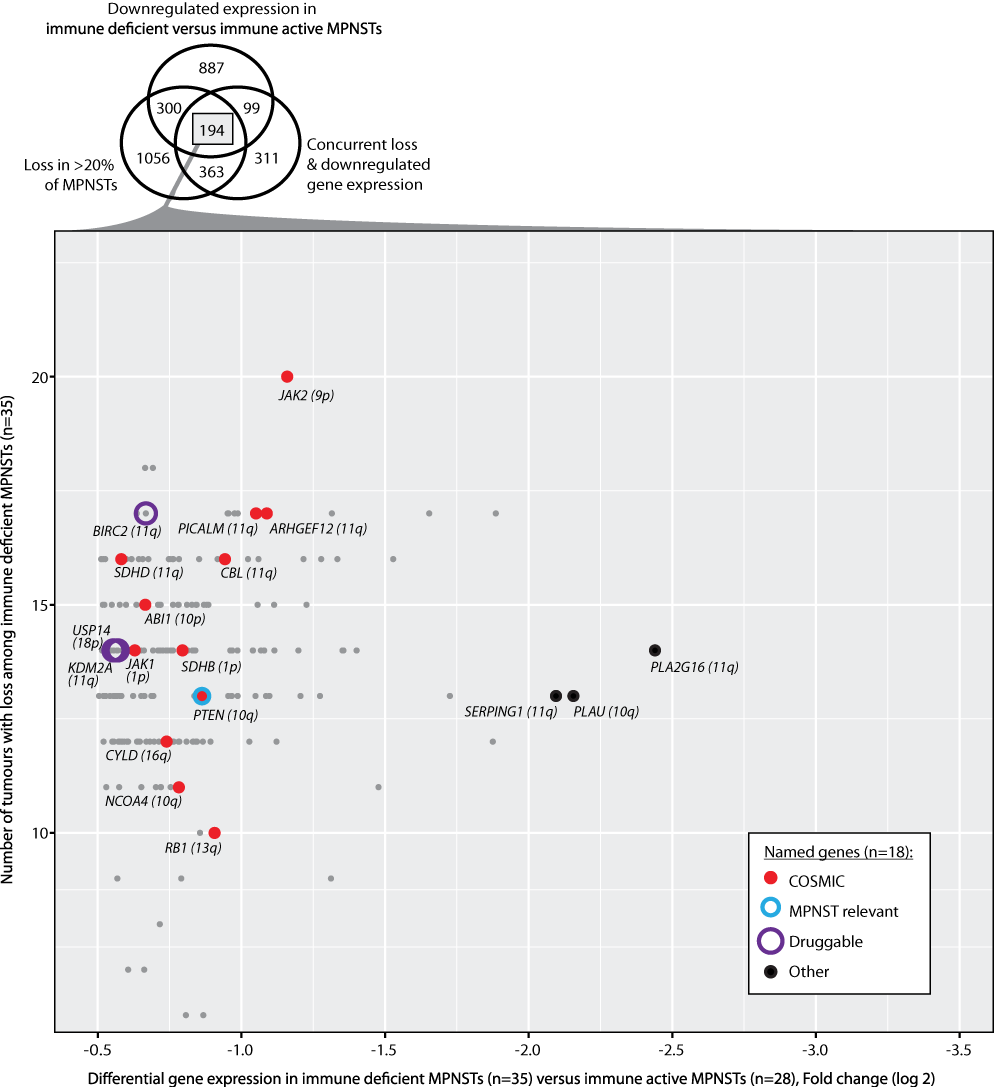


# Supplementary Fig. 12. Downregulated gene expression associated with copy number loss in MPNSTs

Venn diagram of genes (n=194) with downregulated expression in immune deficient (n=35) versus immune active MPNSTs (n=28), loss in > 20% of MPNSTs (scored among all 93 tumors), and with concurrent loss and downregulated gene expression (analyzed in 63 tumours). Cancer-critical genes with downregulated expression driven by copy number loss are highlighted in the scatter plot.


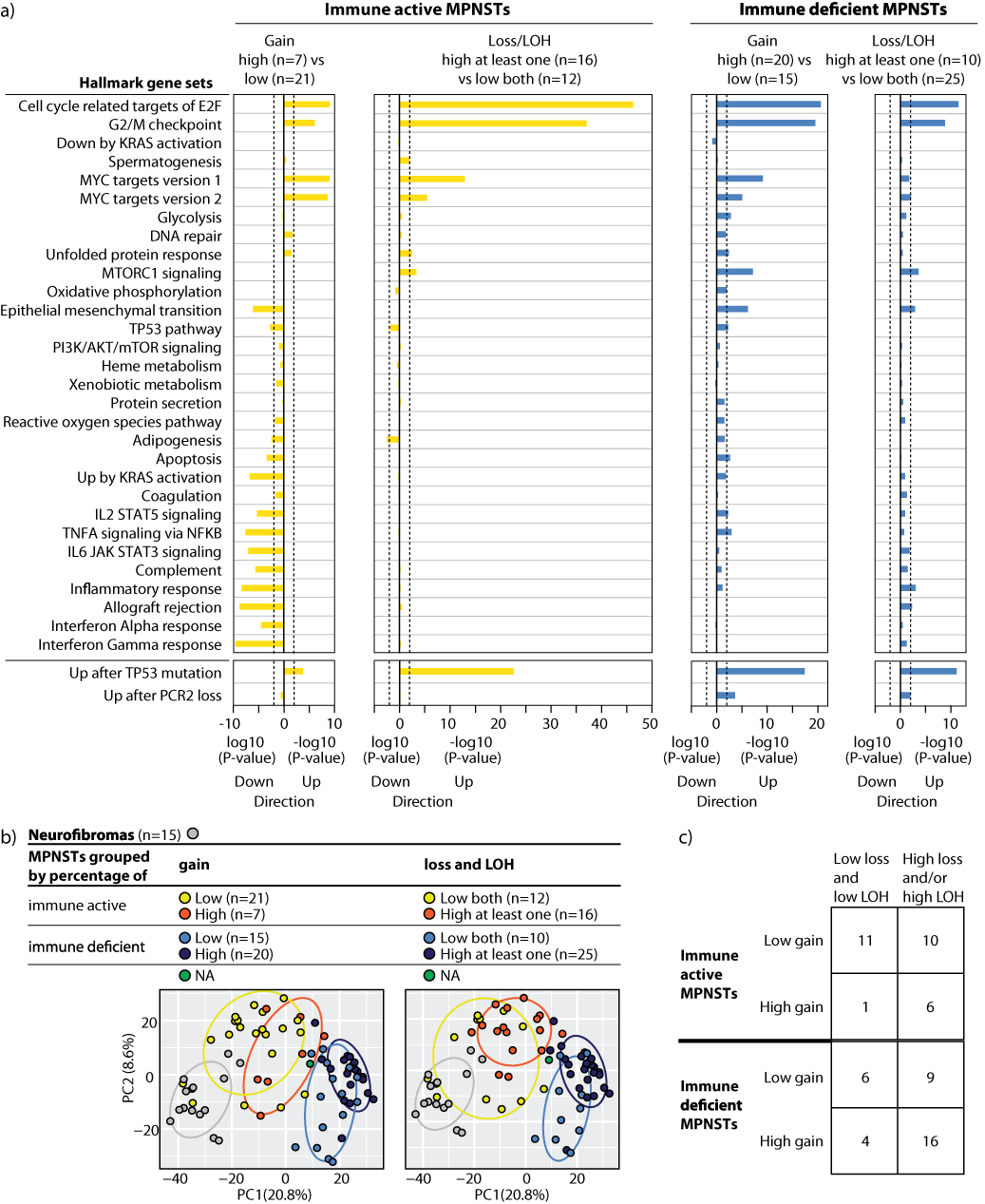


# Supplementary Fig. 13. Gene expression of MPNSTs grouped according to the tumour burden of copy number aberrations

**(a)** Gene set enrichment analyses with the Hallmark (n=50), TP53, and PRC2 gene sets for immune active and immune deficient MPNSTs separately, comparing tumours with high versus low levels of gain or loss/LOH across the genome (thresholds for high and low aberrations levels are given in Figure S8). Gene sets are sorted according to Fig. 2. For the Hallmark gene sets, *P*-values were adjusted by FDR. **(b)** Principal components analysis of MPNSTs and neurofibromas based on genes (n=2388) with variance > 1 among MPNSTs and coloured according to the burden of copy number gain (left) or loss and LOH (right). **(c)** Number of immune active and immune deficient MPNSTs in each copy number category.


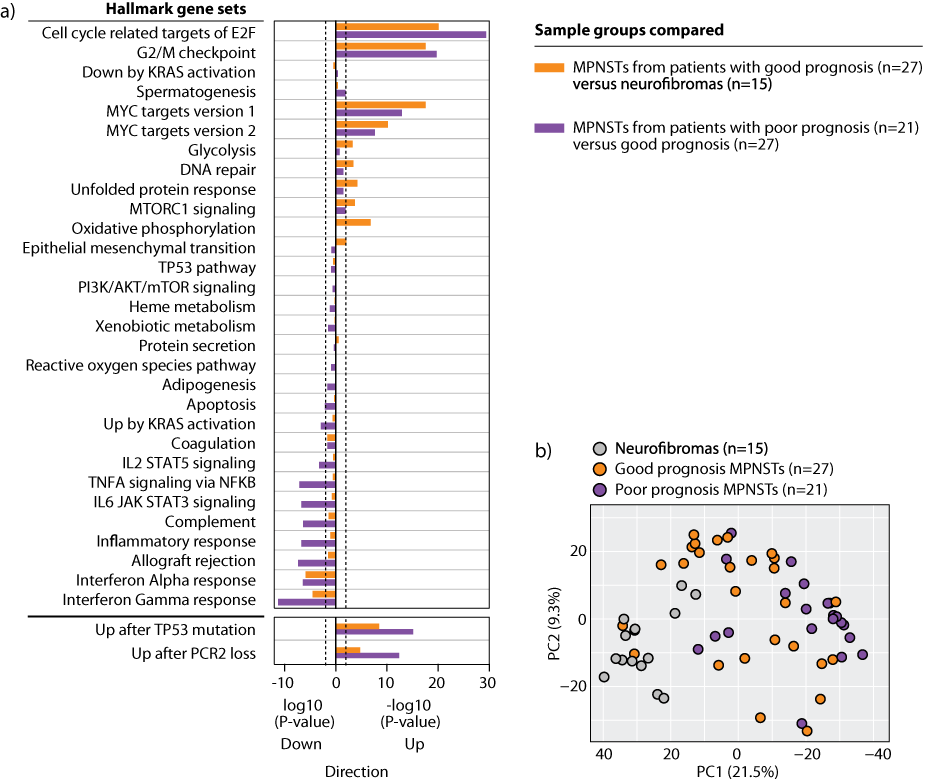


# Supplementary Fig. 14. Gene set enrichments of neurofibromas and MPNSTs according to prognostic group

**(a)** Gene set enrichment analyses with the Hallmark (n=50), TP53, and PRC2 gene sets for immune active and immune deficient MPNSTs separately, comparing MPNSTs from patients with good prognosis versus neurofibromas (in orange) and with MPNSTs from patients with poor prognosis versus good prognosis (in purple). For the Hallmark gene sets, *P*-values were adjusted by FDR. Gene sets are sorted according to Fig. 2, and only gene sets with FDR-*P*<0.01 between MPNSTs from patients with poor prognosis versus good prognosis were included (except the myogenesis gene set, FDR-*P*=0.003). **(b)** Principal components analysis of MPNSTs and neurofibromas based on genes (n=2388) with variance > 1 among MPNSTs and coloured according to prognosis group.


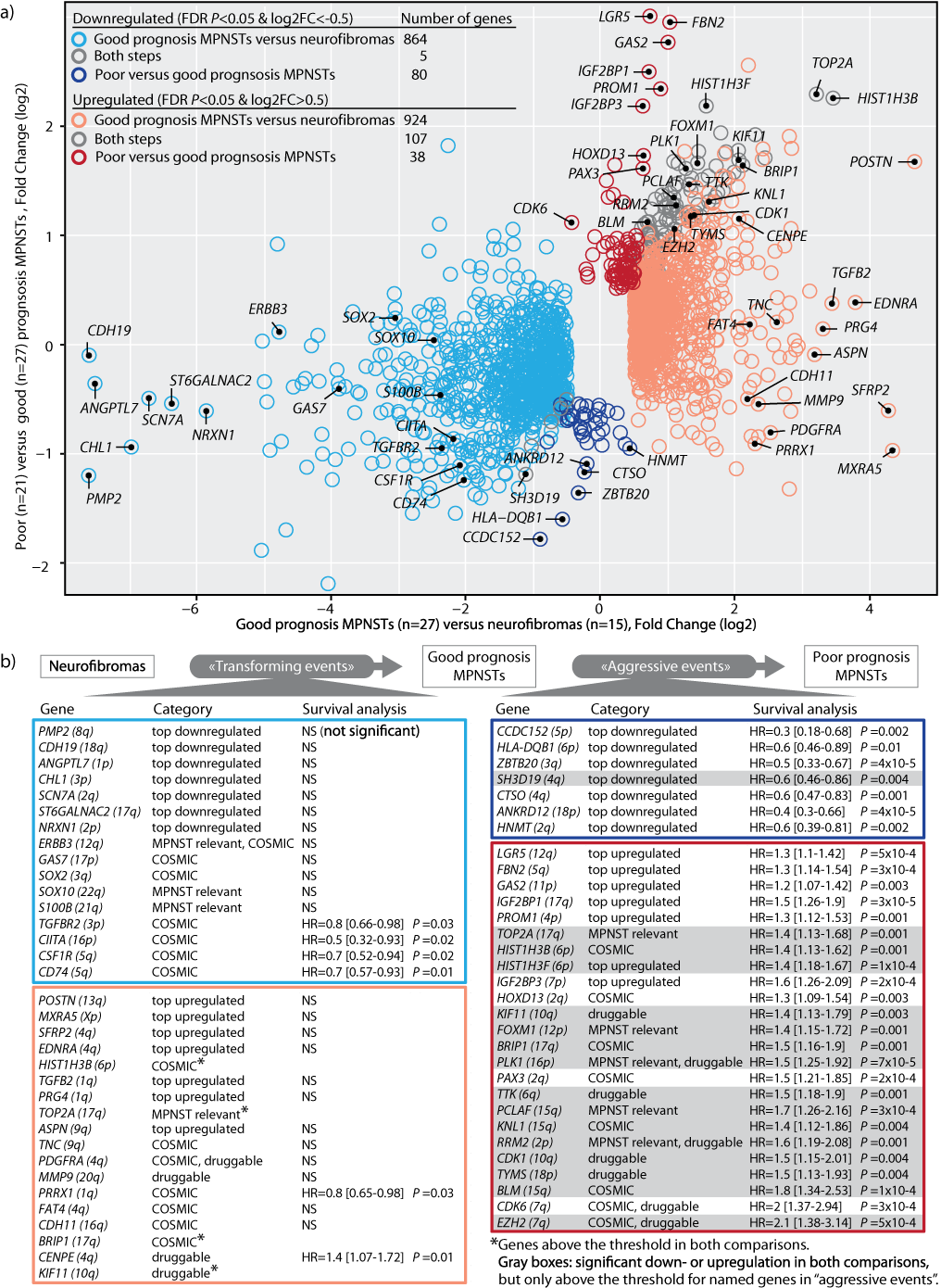


# Supplementary Fig. 15. Differential gene expression between neurofibromas and MPNSTs according to prognostic group

**(a)** Fold change (FC) from analysis of differential gene expression plotted for MPNSTs from patients with poor prognosis versus good prognosis (vertical axis) relative to patients with good prognosis versus neurofibromas (horizontal axis). Selected genes were annotated, including cancer-critical genes (MPNST-related, COSMIC, or upregulated drugable genes), differentially expressed genes in good prognosis MPNSTs versus neurofibromas (log2FC < -2 or > 2 and FDR-*P*<0.05, n=20), differentially expressed genes in poor versus good prognosis MPNSTs (log2FC < -1 or > 1 and FDR-*P*<0.05, n=17 genes), and the seven most up- or downregulated genes. **(b)** Overview of the genes indicated in panel (a) and grouped according to the differential expression analyses (blue indicate downregulated expression and red upregulated expression). Hazard ratios (HRs) and 95% confidence intervals (in brackets) were from Cox regression with *P*-values from Wald test.
